# fMRI signals of pattern separation in the neocortex and hippocampus to non-meaningful objects and their spatial location

**DOI:** 10.1162/IMAG.a.1088

**Published:** 2026-01-16

**Authors:** Zsuzsanna Nemecz, István Homolya, Alex Ilyés, Hunor Kis, György Mező, Virág Anna Varga, Markus Werkle-Bergner, Attila Keresztes

**Affiliations:** Doctoral School of Psychology, ELTE Eötvös Loránd University, Budapest, Hungary; Institute of Psychology, ELTE Eötvös Loránd University, Budapest, Hungary; Brain Imaging Centre, HUN-REN Research Centre for Natural Sciences, Budapest, Hungary; Budapest University of Technology and Economics, Budapest, Hungary; Wigner Datacenter, HUN-REN Wigner Research Centre for Physics, Budapest, Hungary; Konkoly Observatory, HUN-REN Research Centre for Astronomy and Earth Sciences, Budapest, Hungary; MTA Centre of Excellence, HUN-REN Research Centre for Astronomy and Earth Sciences, Budapest, Hungary; Center for Lifespan Psychology, Max Planck Institute for Human Development, Berlin, Germany

**Keywords:** pattern separation, hippocampal subfields, interference resolution, frontoparietal, perirhinal cortex, parahippocampal cortex

## Abstract

Computational theories of memory posit that the dentate gyrus and CA3 (CA3DG) hippocampal subfields reduce mnemonic interference via a process called pattern separation. While the CA3DG is viewed as a domain-general pattern separator, the parahippocampal and perirhinal cortices may play a role in content-specific (e.g., spatial or object-related) interference reduction. Recent work suggests that frontal and parietal control areas may allocate resources during mnemonic discrimination, but the evidence is still limited. Moreover, mnemonic discrimination tasks designed for humans almost exclusively use everyday items as stimuli, confounding retrieval processes with pattern separation. To address these challenges, we acquired high-resolution structural images of the medial temporal lobe, and full-brain high-resolution functional MRI data of 39 participants while they studied non-meaningful fractals with varying degrees of interference in either their spatial or object features. We found that the parahippocampal cortex contributes to interference reduction in the spatial domain, while the perirhinal cortex contributes to interference reduction in the object domain. The dorsolateral frontal and parietal regions were recruited during the encoding of interfering stimuli in both object and location domains and displayed strengthened within- and cross-network connectivity in response to interference. Contrary to our expectations, we did not find significantly increased activation in the CA3DG to similar trials relative to repeats, indicating a lack of sensitivity to small differences in interfering stimuli. Altogether, these results are in line with content-specific interference reduction in the medial temporal lobe, possibly orchestrated by frontoparietal regions, but challenge the view of the CA3DG as a universal pattern separator of the human brain.

## 1 Introduction

Decades of animal and human research have implicated the dentate gyrus and CA3 subfields of the hippocampus as the primary mnemonic pattern separator of the mammalian brain (Amer & Davachi, 2023; Leal & Yassa, 2018; Leutgeb et al., 2007; Rolls, 2013; Yassa & Stark, 2011). Neuroanatomical accounts propose that divergent excitatory projections from the entorhinal cortex to a large number of sparsely firing neurons expand the available representational space in the dentate gyrus (Cayco-Gajic & Silver, 2019). Consistent with this idea, rodent research has shown that even small changes in the environment induce decorrelated activity patterns in the dentate gyrus (GoodSmith et al., 2019; Knierim & Neunuebel, 2016; Leutgeb et al., 2007; Neunuebel & Knierim, 2014). Moreover, selectively disrupting dentate gyrus function in rats (Gilbert et al., 2001; Hunsaker et al., 2008; McHugh et al., 2007) leads to impaired performance on tests assumed to require spatial pattern separation.

In humans, mnemonic discrimination tasks and functional magnetic resonance imaging (fMRI) provided evidence that levels of lure-related univariate activity in the hippocampus and CA3DG are different from repeats and comparable with new trials (Bakker et al., 2008; Kirwan & Stark, 2007). The parametric manipulation of lure similarity relative to repeats has led to the description of non-linear increases in activity in the hippocampus (Motley & Kirwan, 2012; Stark et al., 2013; Yassa et al., 2011). More recently, studies using multivariate fMRI analysis corroborated the notion that the hippocampus reduces the representational overlap between interfering inputs (Berron et al., 2016; Chanales et al., 2017; Favila et al., 2016; Kyle et al., 2015; Wanjia et al., 2021). For instance, Berron et al. (2016) used multivoxel pattern analysis on high-resolution 7T fMRI data to show that two slightly altered spatial scenes could be classified based on the activity of the dentate gyrus, but not other hippocampal subfields or MTL regions. Others have demonstrated that the similarity between representations of stimuli with overlapping features declines over the course of learning in the hippocampus, to the point of becoming less similar than for non-overlapping stimuli (Chanales et al., 2017; Favila et al., 2016; Hulbert & Norman, 2015). The latter phenomenon is often termed “repulsion” (Chanales et al., 2021; Wanjia et al., 2021, 2025), and can be considered theoretically distinct from pattern separation, because unlike simple orthogonalization, it predicts an increase in output dissimilarity with an increase of input similarity (Wanjia et al., 2025). For a deeper discussion of multivariate neuroimaging evidence on the stability, differentiation, and integration of hippocampal representations, see Brunec et al. (2020).

An important and often overlooked confound is that tests of mnemonic discrimination rely on everyday objects or a varying arrangement of these in spatial scenes. Images of everyday objects could induce retrieval of already existing memory representations and confound pattern separation with pattern completion (i.e., the reactivation of the original representation based on a partial or degraded cue) or retrieval processes (Liu et al., 2016). Relatedly, pattern separation measures based on the discrimination of everyday objects cannot differentiate between the active orthogonalization of incoming inputs (i.e., true pattern separation) and disparate voxel activity patterns due to pre-existing, partially overlapping engrams (Quiroga, 2020). Moreover, a substantial part of neuroimaging evidence for pattern separation in the hippocampus comes from recognition tasks, where pattern completion processes may have a larger weight relative to pattern separation. It has been previously argued that pattern separation is a key process in memory formation, and thus it should be ideally studied during encoding (Hunsaker & Kesner, 2013; Liu et al., 2016). Using never-before-seen, non-meaningful stimuli and investigating pattern separation signals at the time of encoding could mitigate these concerns by limiting the activation of already available memories and prior associations.

Recent work also raises the question how extra-hippocampal areas contribute to performance in mnemonic discrimination tasks (Amer & Davachi, 2023; Klippenstein et al., 2020; Nash et al., 2021). According to the recent cortical–hippocampal pattern separation framework, frontoparietal regions could play a key role in directing attention to unique features (Amer & Davachi, 2023), and amplifying the separation of memory representations in the medial temporal lobe (MTL). In turn, cortical MTL areas may be engaged in domain-selective interference reduction. Specifically, the perirhinal cortex (PRC) is implicated in processing and discriminating objects (Barense et al., 2010; Berron et al., 2018; Burke et al., 2014; Bussey et al., 2002; Eacott & Gaffan, 2005; Reagh & Yassa, 2014), while the parahippocampal cortex (PHC) is implicated in processing spatial relations and resolving spatial–contextual interference (Berron et al., 2018; Eacott & Gaffan, 2005; Reagh & Yassa, 2014; Staresina et al., 2011). However, the degree of domain selectivity is debated (Burke et al., 2018; Nilssen et al., 2019), and some studies employing visual and mnemonic discrimination designs did not find such domain specificity (Azab et al., 2014; Lawrence et al., 2020).

The goal of the current study was to assess domain-specific interference reduction in the PHC, PRC, and hippocampus using complex unfamiliar fractals as stimuli in order to reduce the confounding effects of semantic knowledge and retrieval processes. We hypothesized that the PRC will be involved in Object Encoding, the PHC will be involved in spatial encoding, and the CA3DG will be engaged in interference reduction irrespective of the domain. Further, we conducted a whole-brain analysis followed up by exploratory seed-based connectivity analysis to investigate which additional areas are involved in interference reduction of similar stimuli. Although this analysis was exploratory, we expected—based on the cortical–hippocampal pattern separation framework (Amer & Davachi, 2023)—that dorsolateral and parietal regions would be activated by both object and spatial interference, and their connectivity would be modulated by interfering stimuli.

## 2 Methods

### 2.1 Participants

Power analysis based on pilot behavioral data and conducted with the software G*Power (Faul et al., 2007) indicated a sample size of 40 participants needed to show the behavioral effects of the similarity manipulations at 95% statistical power. Forty-six healthy volunteers (25 female, 21 male, 20–30 years old) were recruited for the study from the local student community. All participants were screened for psychiatric or physical illness, metal implants, and current medications. The study was approved by the Ethical Committee of the Medical Research Council, Hungary (ethics approval number: OGYÉI/28291-2/2020), and was carried out in accordance with the code of ethics for human experiments (Declaration of Helsinki). Participants gave written informed consent, and they received either monetary compensation or course credit for their participation. Data of five participants could not be used for the analysis because of technical issues in the MRI (n = 3), unfinished data acquisition (n = 1), and missing log files due to experimenter error (n = 1). Two participants were excluded due to incidental neurological findings. The final sample included 39 participants (19 females, 20 males, 20–30 years old with mean age of 22.61 years and standard deviation of 2.36 years).

### 2.2 Stimuli

**Fig. 1.**
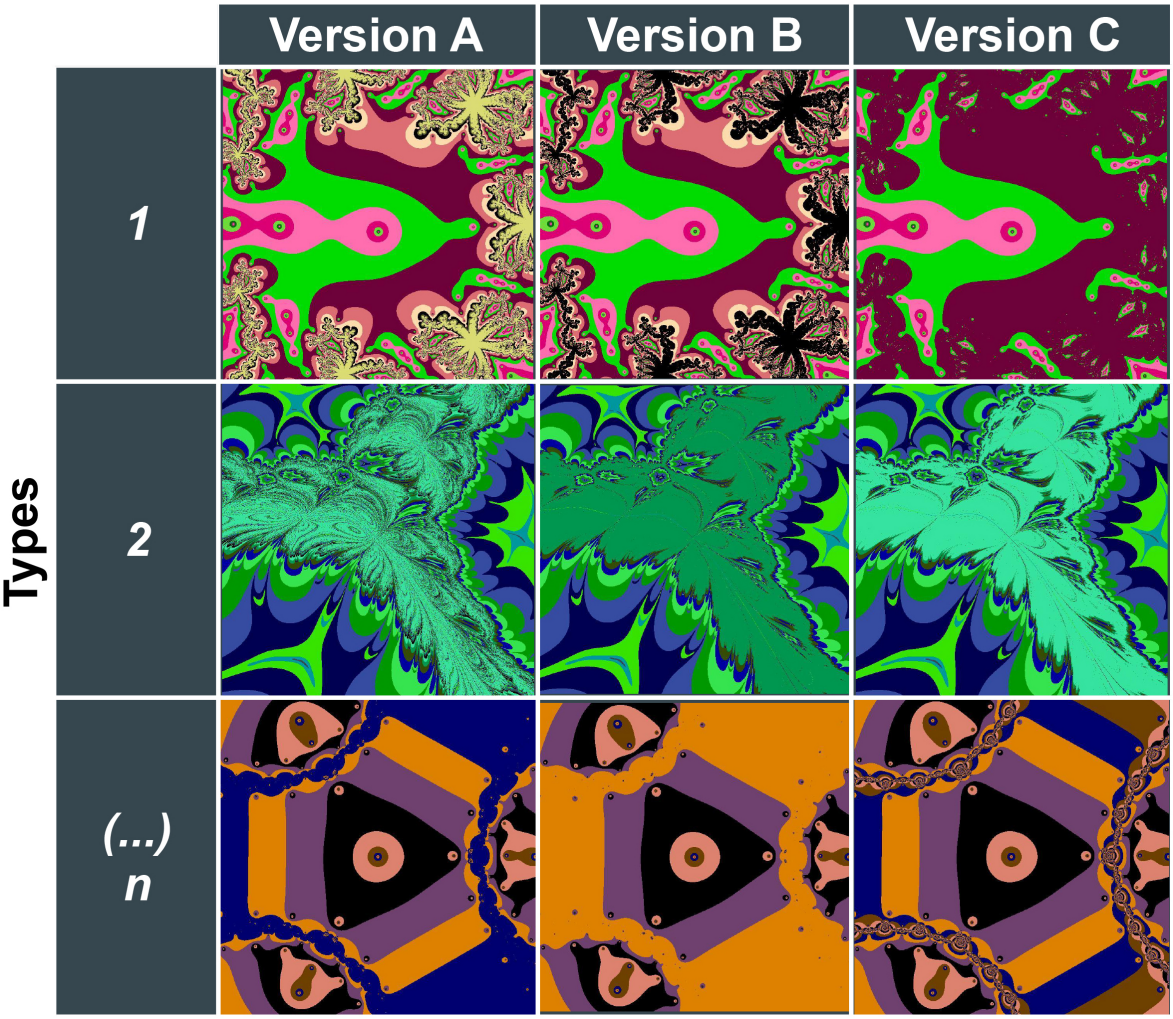
Example fractal stimulus types. Fractals were generated by applying Newton’s method to polynomial and transcendental functions. Fractal types were defined by a unique function and a random color assignment. Three versions of each fractal type—defined by the number of iterations applied—were selected into the stimuli set.

Fractal images (in size 300 × 300 pixels) were generated using Python 3.7.3 by applying the Newton–Raphson method to finding the roots of polynomials and transcendental functions. We created 181 fractal types, each defined by a unique function, and 3 versions of each fractal type (Fig. 1), defined by the number of iterations applied, resulting in a stimuli set of 543. Eleven fractal types were used as demonstration and in practice trials, 1 type was used as a baseline trial that occurred 8 times across tasks, 72 types were randomly assigned to the Location Task, and 96 were randomly assigned to the Object Task. Of the 96 fractal types for the Object Task, 72 were selected for encoding and 24 served as foils during retrieval (see the detailed description below). Fractal images used in the experiment are available here: https://github.com/zs-nemecz/TerKepEsz/tree/MRI_version/stimuli/fractals. Additionally, an art gallery framing was created by combining drawn images of furniture and plants freely available on the internet.

### 2.3 Experimental design

We used two mnemonic discrimination tasks (the Object Task and the Location Task) with either item or spatial similarity manipulation to test content-dependent interference reduction in the medial temporal lobe. The tasks were created in PsychoPy 2021.1.4 (Peirce et al., 2019). Each task consisted of an encoding and a recognition phase, divided into four scanner runs per task (two encoding runs and two recognition runs in each task). During both tasks, a two-dimensional representation of the furnished interior of an art gallery was shown to participants, which contained an empty inner rectangle (1000 by 800 pixels). The location of fractals in both tasks was restricted to the inner rectangle. We used such a framing to enhance the cover story of the task instructions—that participants are sorting and arranging artwork for an exhibition—and to help contextualize spatial locations in the Location Task.

Each trial consisted of a fractal appearing in the inner rectangle of the screen. Trial duration was 3 s during the encoding phase, and 4 s during the recognition phase. All trials were preceded by a fixation cross, which was presented at the center of the consecutive fractal’s location for a jittered interval between 0.5 and 8 s.

After each encoding run and preceding the recognition run, participants had to solve a simple mathematical problem to reduce recency effects, which was followed by a short break. The delay between the last encoding trial and first recognition trial was around 2 min on average. Participants were aware of the memory manipulation already during encoding, and they were instructed before the recognition phase to pay attention to small changes of fractals in the Object Task, and small changes of location in the Location Task.

### 2.4 Object task

**Fig. 2.**
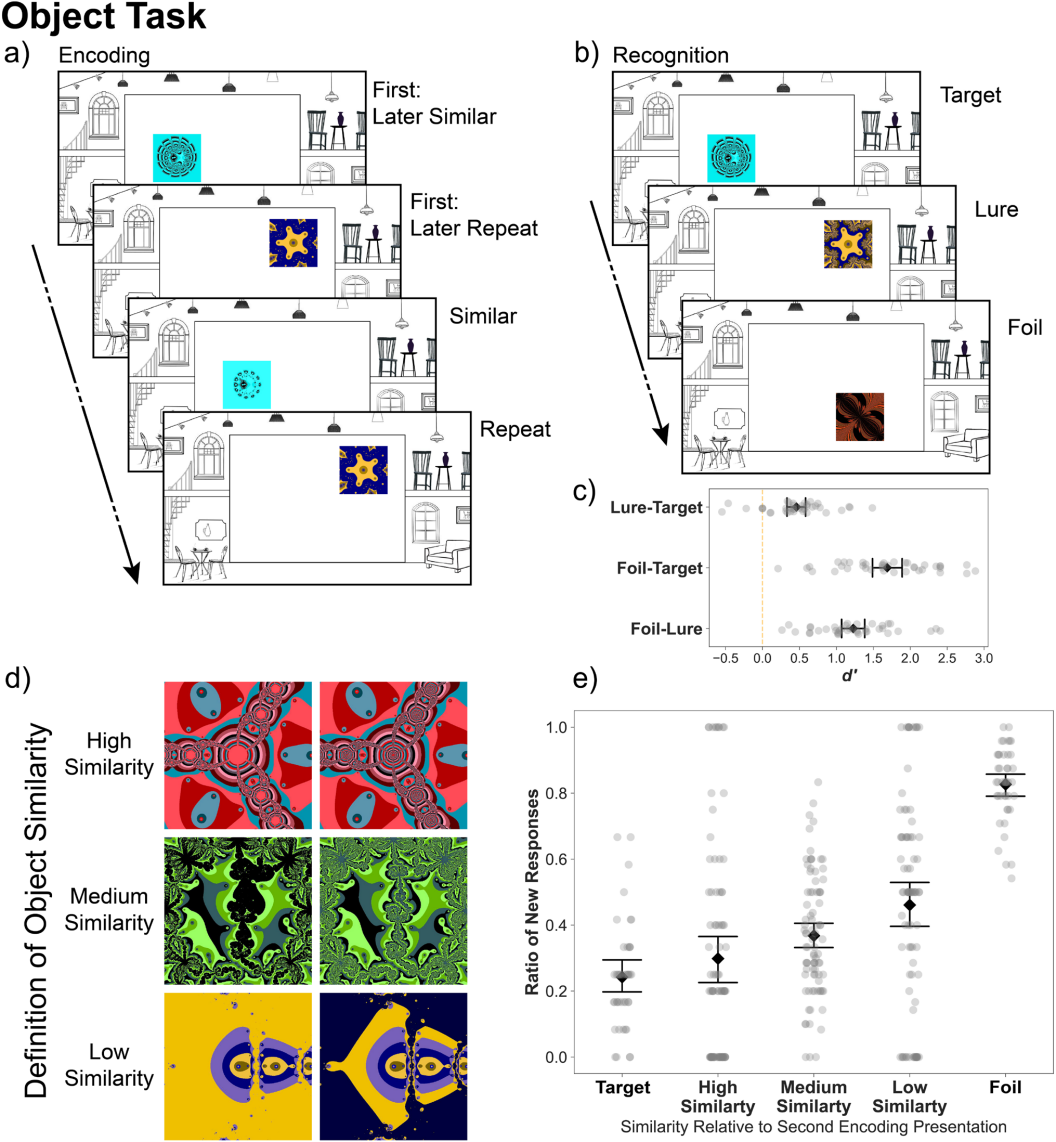
Object Task. Design, procedure, and recognition performance. (a) The structure of the Object Task. During encoding, participants saw fractal images appear in an art gallery context (constant through the experiment) and were instructed to make esthetic judgments about the images. Each trial lasted 3 s, with jittered inter-trial intervals (0.5–8 s). (b) During recognition, participants had to judge whether they had seen exactly this fractal in the previous encoding phase. Trial duration was 4 s with jittered inter-trial intervals (0.5–8 s). (c) Discriminability of each condition in the Object recognition phase. (d) Example pairs from each similarity bin used for the behavioral analysis. An independent sample of 30 participants rated each fractal pair on a scale of 0–9 (Identical - Very Different). (e) The ratio of “new” responses is dependent on the similarity of lure fractals. The × axis depicts the similarity between the first presentation of the fractal type and the version presented at recognition. The y axis depicts the raw ratio of “new” responses for each participant. Error bars represent 95% confidence intervals.

The Object Task structure is depicted in Figure 2. An overview of stimuli assignment in the Object Task is shown in Supplementary Figure S5.

#### 2.4.1 Encoding phase

In the Object Encoding phase (Fig. 2a), participants made binary esthetic judgments about each fractal, answering the following question: “Would you like to display this image in the exhibition of the art gallery?” This question served two purposes. First, we wanted to make sure participants attend to the features of each fractal. Second, although in principle passive viewing could have elicited our neural signals of interest, by requiring a response from participants, we could make sure that they pay attention throughout the task. For encoding, we selected 72 fractal types that were shown in one of the three possible versions (VER_1st_) at their first appearance at a randomly assigned location (LOC_1st_). We refer to those first presentations as new stimuli at encoding (NEW_ENC_). From those 72 fractal types, 24 were repeated during encoding in the same version at exactly the same location (LOC_1st_). We refer to those second presentations as repeats during encoding (REP_ENC_). The remaining 48 fractal types were also shown a second time during encoding at the same location (LOC_1st_), but in a different version (VER_2nd_). We refer to those second presentations as similar during encoding (SIM_ENC_). We used twice as many similar items as repeats to increase the power of detecting differences between similarity bins, and small increases relative to repeats, assuming that repetition suppression is a robust effect that may be detectable with less trials than the sensitivity to similarity. The lag between first (NEW_ENC_) and second presentations (SIM_ENC_ and REP_ENC_ trials) was jittered between 2 and 6 trials.

#### 2.4.2 Recognition phase

In the Object Recognition phase (Fig. 2b), participants had to make “old”/”new” memory judgments, corresponding to the following question: “Have you seen exactly this image before?” Twenty-four of the recognition trials were targets (same fractal at the same location as the original), 24 were lures (another version of the same fractal type, presented at the same location as the original), and 24 were foils (new fractal at a randomly assigned location). For the recognition phase, we selected 48 fractal types from the first presentations during encoding (i.e., NEW_ENC_). Half of the fractal types, that is, 24, became target items during recognition (TARGET_REC_). The other half, that is, 24, was used as lure items during recognition (LURE_REC_). During recognition, TARGET_REC_ items were always shown in the exact same version they appeared during the first presentation at encoding (i.e., VER_1st_). The 24 TARGET_REC_ items were selected in a way that 12 have been repeats during their second presentation at encoding (i.e., REP_ENC_), while the remaining 12 have been shown in a similar version during their second presentation at encoding (i.e., SIM_ENC_). The LURE_REC_ items were always shown in the fractal version of the three possible ones that was not yet used in the task, so far (i.e., VER_3rd_). Similar to the target items, also the 24 LURE_REC_ items were selected in a way that 12 have been shown as exact repeats (i.e., REP_ENC_) and 12 as similar versions (i.e., SIM_ENC_) during their second presentation at encoding. The 24 target and 24 lure items were intermixed with 24 foil items (FOIL_REC_). The FOIL_REC_ items were selected from novel fractal types that were not presented during encoding. The version of FOIL_REC_ fractals was randomly drawn from the three possible versions (i.e., VER_foil_).

#### 2.4.3 Object similarity manipulation

Similar (SIM_ENC_) and lure (LURE_REC_) fractals in the Object Task were different versions of the same fractal type as the original fractal presented during encoding (VER_1st_, Fig. 1), that is, the fractal was created using the same function but a different number of iterations. The similarity between fractal versions was rated post hoc by an independent sample of participants (n = 30) on a scale from 0 (“Same”) to 9 (“Very Different”). Details of the rating experiment and the distribution of fractal similarity values are included in the Supplementary Materials. Figure 2d shows examples of fractal pairs with high, medium, and low similarity ratings.

### 2.5 Location task

**Fig. 3.**
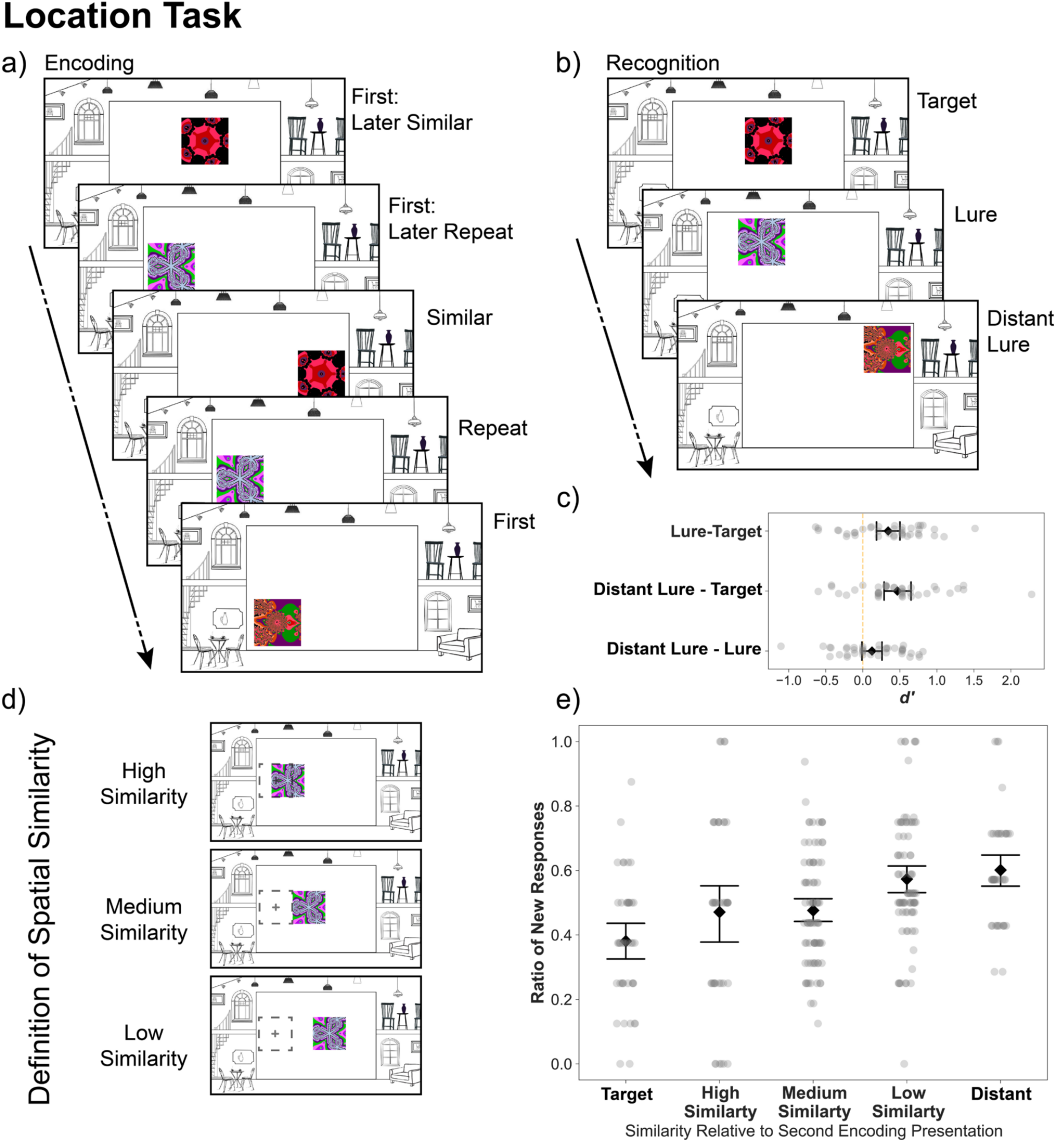
Location Task. Design, procedure, and recognition performance. (a) The structure of the Location Task. During location encoding, participants saw fractal images appear in an art gallery context (constant through the experiment) and were instructed to make esthetic judgments about the position of the images. Each trial lasted 3 s, with jittered inter-trial intervals (0.5-8 s). (b) During recognition, participants had to judge whether they had seen the shown fractal at the exact current location in the preceding encoding phase. Trial duration was 4 s with jittered inter-trial intervals (0.5-8 s). (c) Discriminability of each condition in the Location Recognition phase. (d) Example pairs from each similarity bin used for the behavioral analysis. Very close pairs were presented 1–200 pixels away, close pairs were presented 200–400 pixels away, far pairs were presented 400–600 pixels away from the second presentation. Very far pairs are more than 600 pixels away from the second presentation. (e) The ratio of “new” responses is modulated by distance. The × axis depicts the spatial similarity between the second presentation of the trial and the recognition trial. The y axis depicts the raw ratio of “new” responses for each participant. Error bars represent 95% confidence intervals.

The Location Task structure is depicted in Figure 3. An overview of stimuli assignment in the Location Task is shown in Supplementary Figure S6.

#### 2.5.1 Encoding phase

The encoding phase of the Location Task (Fig. 3a) was analogous to the encoding phase of the Object Task, but here similar trials were defined by their Euclidean distance to the first presentation of the fractal, not their within-item features. Participants made binary esthetic judgments about the location of each fractal image. Specifically, participants had to decide whether they would like to display the presented image at the current location in the art gallery, which encouraged participants to pay attention to the location of each fractal. For encoding, we selected 72 fractal types that were shown in one of the three possible versions (VER_1st_) at their first appearance at a randomly assigned location (LOC_1st_). We refer to those first presentations as new stimuli at encoding (NEW_ENC_). From those 72 trials, 23 were repeated during encoding in the same version (VER_1st_) at exactly the same location (LOC_1st_). We refer to those second presentations as repeats during encoding (REP_ENC_). The remaining 49 fractals were also shown a second time during encoding in the same fractal version (VER_1st_), but at an alternative location (LOC_2nd_). We refer to those second presentations as similar during encoding (SIM_ENC_). Locations were unique to each NEW_ENC_ and SIM_ENC_ trial, thus there were altogether 120 locations used in the Location Encoding phase. Trial order was pseudo-randomized, and the lag between first (NEW_ENC_ trials) and second presentations (SIM_ENC_ and REP_ENC_ trials) was jittered between one to six trials.

#### 2.5.2 Recognition phase

During the Location Recognition phase (Fig. 3b), fractals were presented again in the inner rectangle of the screen, and participants had to make “old”/”new” memory judgments, corresponding to the following question: “Have you seen this image exactly at this location?” Participants were made aware of the fact that only fractals shown in the previous encoding phase were presented during recognition. For the recognition phase, we used all 72 first presentation trials from the encoding phase (i.e., NEW_ENC_). One-third of these trials, that is, 24, was used as target trials (TARGET_REC_), one-third served as the basis for lure trials (LURE_REC_), and one-third for distant lure trials (DISTANT_REC_). TARGET_REC_ trials were always shown in the exact same fractal version and exact same location as they appeared during the first presentation at encoding (i.e., VER_1st_ LOC_1st_). The 24 TARGET_REC_ trials were selected in a way that 8 have been repeats during their second presentation at encoding (i.e., REP_ENC_), while the remaining 16 have been shown at a similar location during their second presentation at encoding (i.e., SIM_ENC_). The LURE_REC_ trials were never presented in an exact fashion during encoding, but the same fractal (VER_1st_) was presented at a different location during encoding (i.e., LOC_3rd_). Similar to the target items, also the 24 lure items were selected in a way that 8 have been shown as repeats (i.e., REP_ENC_) and 16 as similar (i.e., SIM_ENC_) during their second presentation at encoding. The distant lure trials (DISTANT_REC_) were VER_1st_ fractals presented in a location at least 450 pixels away (LOC_DIST_) from the first presentation location (LOC_1st_). TARGET_REC_, LURE_REC_, and DISTANT_REC_ trials were intermixed and their order was pseudorandomized.

#### 2.5.3 Spatial similarity manipulation

During the encoding phase of the Location Task, similar trials (SIM_ENC_) consisted of repeated (VER_1st_) fractals presented 200 to 350 pixels away from their original location (LOC_2nd_). The exact distance was randomly sampled from a uniform distribution.

For the recognition phase, we originally grouped trials into target (TARGET_REC_), lure (LURE_REC_), and distant lure (DISTANT_REC_) conditions based on their Euclidean distance to the very first presentation of the fractal in the encoding phase (LOC_1st_). Twenty-four of the recognition trials were thus exactly repeated, 24 were lures separated by at least 200 and maximum 350 pixels, and 24 were distant lures separated by at least 450 pixels. However, each trial’s spatial similarity can also be calculated based on the Euclidean distance to the second presentation of the fractal during encoding, which also affected participants’ “new” responses (Fig. 3e). Distributions of lure and distant lure distances to the first and second presentation can be found in the Supplementary Materials (Supplementary Fig. S7).

### 2.6 Experimental procedure

All participants completed both the Location and the Object Tasks. The order of the tasks was counterbalanced across participants. Participants were taken out of the scanner between the tasks for a longer break, and they were given the task-specific instructions immediately preceding the task. Before each task, participants were given six practice trials with feedback. Encoding and recognition phases were divided into two runs each, and participants completed them in an interleaved fashion, so that a recognition run followed each encoding run. For example, a participant starting with the Object Task had the following procedure: Object Task Instructions and Practice, Object Encoding 1, Object Recognition 1, Self-paced break in the scanner, Object Encoding 2, Object Recognition 2, Long break outside the scanner, Location Task Instructions and Practice, Location Encoding 1, Location Recognition 1, Self-paced break in the scanner, Location Encoding 2, Location Recognition 2.

### 2.7 Behavioral data analysis

We analyzed the ratio of “new” responses with a one-way repeated-measures ANOVA with the within factor condition (target, lure, foil) in Python 3.9.13 with statsmodels 0.13.2 (Seabold & Perktold, 2010). Follow-up paired sample t-tests were conducted with scipy 1.13.1 (Virtanen et al., 2020), and we controlled for the false discovery rate (FDR) with the Benjamini–Hochberg method (Benjamini & Hochberg, 1995). Figures were created with matplotlib 3.9.2 (Hunter, 2007) and seaborn 0.13.0 (Waskom, 2021).

To further assess the effect of similarity per trial, we used generalized binomial linear mixed effects modeling implemented in the R package lme4 1.1-31 (Bates et al., 2015). For each task, we built three models, predicting the probability of a “new” response per recognition trial. Assumption checks for the mixed effects models are described in the Supplementary Materials (Supplementary Methods section).

The first model (Model A) included the similarity between the first encoding trial and the recognition trial as a fixed effect predictor, and the participant-specific intercept as a random effects predictor. The second model (Model B) included the similarity between the second encoding trial and the recognition trial as a fixed effect predictor, and the participant-specific intercept as a random effects predictor. The third model (Model C) included the similarity between both the first and the second encoding trial and the recognition trial as fixed effect predictors, and the participant-specific intercept as a random effects predictor. Accordingly, for each task, the three models were defined as follows:

Model A: *RespNew ~ FirstSimilarity* *+* *(1 | Participant)*,
Model B: *RespNew ~ SecondSimilarity* *+* *(1 | Participant)*,
Model C: RespNew ~ FirstSimilarity * SecondSimilarity *+* *(1 | Participant)*,

where *RespNew* is the probability of a “new” response, *FirstSimilarity* is the similarity between the first encoding trial and the recognition trial, and *SecondSimilarity* is the similarity between the second encoding trial and the recognition trial.

For the correlations between behavior and neural measures, we obtained the measures of individual sensitivity to similarity from modified versions of models A and B, which, in addition to the random intercepts, included random slopes. This type of model allows for the relationship between the explanatory variable (here: similarity) and the predicted variable (here: new response) to be different for each individual. Participant-specific coefficients (individual slopes) were then extracted from the models.

### 2.8 MRI data acquisition

A Siemens Magnetom Prisma 3T MRI scanner (Siemens Healthcare, Erlangen, Germany) with the standard Siemens 32-channel head coil was used. Functional measurements were acquired with a conventional gradient-echo-EPI sequence using multiband-acceleration with factor 4 (80 slices, slice gap: 0.375 mm, slice ordering: interleaved, phase encoding direction: anterior to posterior, repetition time (TR) = 1840 ms, echo time (TE) = 30 ms, flip angle (FA) = 72°; field of view (FOV) = 204 mm, spatial resolution 1.5 × 1.5 × 1.5 mm). Fieldmaps for the functional runs were acquired each time the participant was placed in the scanner, resulting in one fieldmap per task. We obtained sagittal T1-weighted images using a 3D MPRAGE sequence (TR = 2300 ms,TE = 3.03 ms, FA = 9°, FOV = 256 mm, spatial resolution 1 × 1 × 1 mm). An additional restricted FOV high-resolution proton-density (PD)-weighted image of the medial temporal lobe area was acquired (TR = 6500 ms, TE = 16 ms, FA = 120°; spatial resolution 0.4 × 0.4 × 2 mm, FOV = 206 mm) to aid the delineation of the anatomical regions of interest.

### 2.9 Subject-level ROI analysis

Only data from the encoding runs are analyzed here. Functional data preprocessing and analyses were done using FSL FEAT (Jenkinson et al., 2012; Woolrich et al., 2001, 2004, 2009). Functional runs were undistorted using fieldmaps and the T1 structural image. Then, each slice was spatially realigned to the middle slice within each volume with FSL MCFLIRT (Jenkinson et al., 2002), resulting in motion parameters that were used as regressors in further analysis for motion correction. As we strived to retain as much individual anatomical specificity as possible, functional runs were not transformed to the T1 or standard space for the region of interest analysis (see section “Registration of Anatomical Regions of Interest” below). In order to enable the analysis of multiple runs per subject in a single higher-level model, we used affine registration with six degrees of freedom to transform each run per participant to the same participant-specific functional space. To take advantage of the high spatial resolution functional scans, and considering the small size of hippocampal subfields, we applied minimal smoothing on the data with a 2-mm full-width at half-maximum isotropic Gaussian kernel. Finally, we applied a high-pass filter with a threshold of 90 s on functional runs. The threshold of 90 s was estimated by FSL Feat based on the trial timing.

For each participant, we performed two general linear model analyses using the data of the encoding phase. Regressors were defined by convolving the canonical hemodynamic response function with a boxcar function representing the onsets and duration of the events within each explanatory variable.

**Fig. 4.**
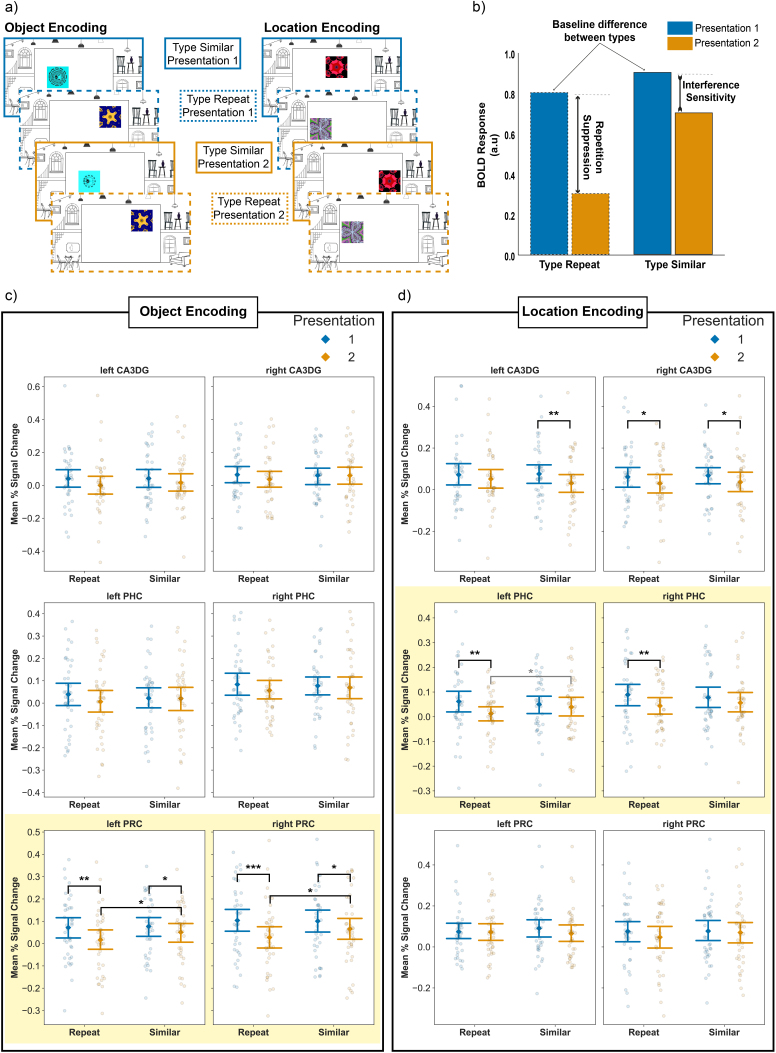
BOLD responses in the encoding phase modeled in a 2-by-2 design. (a) First presentation trials were divided into first presentations of later similar trials and later repeats. (b) Expected results and their interpretation from the 2-by-2 model. (c) Responses of the main medial temporal regions of interest during the Object Encoding. (d) Responses of the main medial temporal regions of interest during Location Encoding. Neural response patterns in (c) and (d) suggesting differentiation between similar and repeat trials are shaded with yellow. Significant differences (unadjusted) are denoted with a symbol: *p < .05, **p < .01, ***p < .001. The comparison between the second presentation repeat and similar trials in the left PHC (panel d, Location Task, marked with a gray *) was not significant after correcting for multiple comparisons. Abbreviations: CA3DG—CA3 and dentate gyrus, PHC—parahippocampal cortex, PRC—perirhinal cortex. Error bars represent 95% confidence intervals.

First, we modeled the new, similar, and repeated encoding trials in a 2 (presentation rank: first vs. second) - by - 2 (trial type: similar vs. repeat) design with four regressors: first presentation of later repeats (Presentation 1, Type Repeat), first presentation of later similar trials (Presentation 1, Type Similar), repeats (Presentation 2, Type Repeat), and similar trials (Presentation 2, Type Similar). The design is depicted in Figure 4a and the interpretation of expected results is depicted in Figure 4b. We opted for this balanced model to ensure that differences between Types are not confounded by potential baseline differences between the first presentations of later repeat and similar trials. In a 2-by-2 repeated measures ANOVA, such an imbalance would be indicated by a main effect of trial type. We found no indication of a baseline difference between trial types, and thus we collapsed all first presentation trials in the follow-up analysis.

In the second model, we collapsed all first presentation trials, and divided similar trials into high-similarity and low-similarity trials. This resulted in four explanatory variables of interest: repeat, high similarity, low similarity, and new (i.e., first presentation) trials.

A single baseline trial was repeated eight times across tasks. The first presentation of this trial was included among the first presentation of later repeat trials (and in the second model, among the merged first presentation trials), the second presentation of this trial was included among repeats, and the third to the eighth presentations were included in a separate explanatory variable in both models. We deemed the number of these multiple repetition trials (third to eighth) too low to include them in further analysis, but exploratory results are shown in Supplementary Figure S2.

In both GLM analyses, we included the six head motion parameters estimated in the realignment procedure and trial-wise reaction times (Mumford et al., 2024) as nuisance regressors. The resulting regressors were fitted to the observed functional time series of the encoding phase. Mean percent signal change values from all regions of interest were extracted from the individual participant space with FSL featquery and were analyzed with Python libraries scipy (Virtanen et al., 2020) and statsmodels (Seabold & Perktold, 2010), and R package lme4 (Bates et al., 2015). All reported *p* values, unless otherwise stated, are FDR adjusted per group of analysis (Benjamini & Hochberg, 1995), to account for multiple comparisons.

### 2.10 Anatomical regions of interest

Anatomical regions of interest (ROIs) in the MTL were delineated on the high-resolution PD images with the software Automatic Segmentation of Hippocampal Subfields (ASHS, Yushkevich et al., 2015). We used the UPENN PMC Atlas (Yushkevich et al., 2015) for the segmentation of the perirhinal and parahippocampal cortices, and a custom atlas (Bender et al., 2018) for the delineation of subfields within the hippocampal body (cornu Ammonis 1–2: CA1, subiculum: SUB, cornu Ammonis 3 and dentate gyrus: CA3DG). Due to the less uniform structure of the hippocampal head and tail, consensus-based, histologically validated segmentation protocols have yet to be established for these regions (Bender et al., 2018; Olsen et al., 2019; Wisse et al., 2017). Therefore, only the hippocampal body was segmented in this study. As in numerous previous high-resolution fMRI studies (Nash et al., 2021; Reagh & Yassa, 2014), the subfields CA3 and DG were combined into a single ROI (CA3DG). The quality of the segmentations was inspected by an experienced rater (A.K.), and errors in the hippocampal body were corrected following the recent guideline by Canada et al. (2023). Briefly, all subfields delineated on all hippocampal body slices were inspected for five error types: lack of label, underestimated label, overestimated label, dropped pixels, missed sulcal cavity. If the errors affected at least 25% of voxels of a given subfield (as estimated by the rater), the error was manually corrected for that subfield within that slice. The number and types of errors that were manually corrected are reported in Supplementary Table S14.

Segmented regions were binarized and used as masks for extracting mean percent signal change values in the univariate and ROI-to-ROI connectivity analysis. An example segmentation is shown in Supplementary Figure S8.

We selected three parietal and frontal areas per hemisphere that emerged consistently across the two tasks during similar lure trials in the whole-brain analysis (the angular gyrus, the supramarginal gyrus, and the middle frontal gyrus) to serve as seeds in the Generalized psychophysiological interaction analysis (gPPI analysis) and ROI-to-ROI connectivity analysis. Cortical parcellation based on the Destrieux atlas (Destrieux et al., 2010; Fischl, 2004) was acquired for each participant using Freesurfer (Dale et al., 1999; Desikan et al., 2006; Fischl et al., 1999, 2002). Freesurfer segmentations were transformed into functional space, and ROI labels were extracted and binarized. Using the ROI masks, we derived the mean time series data from each functional run, and used these as physiological variables. The gPPI models included a regressor for each task condition and the interaction between the seed activity and task condition.

### 2.11 Registration of functional and high-resolution anatomical images

We defined each participant’s individual functional space as the mean volume of the first Object Encoding run. All other functional runs were registered to this volume with rigid body registration as implemented in FSL FLIRT (Jenkinson & Smith, 2001; Jenkinson et al., 2002) during the analysis of fMRI data. Anatomical masks were transformed into functional space with Advanced Normalization Tools (ANTs, Avants et al., 2009, 2011) using a three-step registration procedure. All input images were N4 bias-field corrected before registration. First, we registered the standard T1 anatomical image of each participant to the corresponding high-resolution partial field-of-view PD image with affine transformations, and obtained the transformation matrix *T1-to-PD*. Second, we registered the mean skull-stripped functional volume to the skull-stripped T1 anatomical image first using rigid transformation and then symmetric image normalization (SyN, Avants et al., 2008), and obtained the transformation matrix *meanFunc-to-T1* and warp image *meanFunc-to-T1*. For the meanFunc to T1 registration, we used a generous medial temporal lobe mask to optimize the registration outcome in this region. Exact parameters of the ANTs registration calls as well as registration examples can be found in the Supplementary Materials. Third, we transformed medial temporal ROIs derived from the PD space to functional space by applying the inverse of the *meanFunc-to-T1* matrix, the inverse of the *meanFunc-to-T1* warp, and finally the inverse of the *T1-to-PD* matrix.

### 2.12 Whole-brain group-level fMRI analysis

We generated a group-specific anatomical template with ANTs by registering individual T1 scans to a template space and then averaging them to form a whole-brain image representative of our sample. The average of all original T1 input images was used as the starting point for the iterative registration process. N4 bias field correction was applied on all input images before registration. One linear registration of input files was followed by four nonlinear SyN iterations. Each SyN registration was performed over four increasingly fine-grained resolutions (100 × 100 × 70 × 20 iterations). The similarity metric was cross-correlation. Template update steps were set to 0.2 mm.

The fMRI data processing steps for the whole-brain analysis were identical to those of the subject-level region-of-interest analysis described above, with the exception of registering functional runs to the individual T1 scans with BBR registration implemented in FSL (Greve & Fischl, 2009). Individual T1 scans were normalized to the group template with nonlinear registration to facilitate group-level analysis.

We modeled first, similar, and repeated encoding trials in a 2-by-2 design with four regressors as above (Fig. 4a), separating the first presentation of later similar and of later repeat trials into two regressors. Following previous work (Nash et al., 2021; Reagh & Yassa, 2014), we chose the contrast *similar - first presentation of later similar trials* as our comparison of interest to identify areas that show high activity to similar trials, disregarding the visual features they share with first presentations and tapping on mnemonic processes. We ran fixed-effects analyses to combine the runs within participants, and mixed-effects analysis (FLAME 1) to obtain the group-level results. We used the cluster thresholding option with a Z statistic threshold of 2. Group-level contrast parameter estimate (COPE) images were transformed into MNI152 standard space, after coregistering the group T1 template image to the 1 mm MNI152 template brain available in FLS. We obtained anatomical labels reported in the whole-brain group-level results from the Harvard-Oxford Cortical Atlas (Desikan et al., 2006; Frazier et al., 2005; Goldstein et al., 2007; Makris et al., 2006).

### 2.13 Generalized psychophysiological interaction analysis

Psychophysiological interaction (PPI) analysis examines task-specific modulations of the functional connectivity of an area (Di et al., 2021; Friston et al., 1997) by modeling the interaction between the time series of the area (physiological variable) and a task condition (psychological variable). In a generalized psychophysiological interaction analysis (gPPI), in contrast to the standard PPI approach, each task condition and its interaction with the physiological variable is modeled, thus covering the entire experimental space, leading to better model fit (McLaren et al., 2012). We used three parietal and frontal areas as seeds in the gPPI analysis: the angular gyrus, the supramarginal gyrus, and the middle frontal gyrus. The masks were obtained using the Freesurfer parcellations (see section “Anatomical regions of interest”). We derived the mean time series data from each functional run and seed mask, and used these as physiological variables. The gPPI models included a regressor for each task condition and the interaction between the seed activity and task condition.

### 2.14 ROI-to-ROI connectivity analysis

We extracted the mean preprocessed time series data from each functional run using our medial temporal (left and right CA3DG, PHC, PRC) and frontal (left and right middle frontal gyrus) and parietal (left and right angular gyrus, supramarginal gyrus) masks. The entire time course of each run was used for this analysis. Motion parameters were regressed out of all ROI time series data. Next, we correlated the time series data from each medial temporal region with all frontal and parietal regions, and created one correlation matrix per functional run and subject. We applied Fisher’s z transformation on the correlation coefficients, and then averaged matrices across runs of the same task, thus acquiring one correlation matrix per subject and task. We tested whether there is a significant connectivity between pairs of regions with one-sample t-tests. Connectivity differences between the Location and Object Encoding were tested with paired sample t-tests. The relationship between connectivity and behavior was assessed with Spearman correlations, as we had no predictions on the linearity of the associations. Pearson correlations yielded the same pattern of results, but indicated stronger relationships between variables.

## 3 Results

### 3.1 Behavioral results—Recognition phase

#### 3.1.1 Ratio of “new” responses per condition

A one-way repeated-measures ANOVA revealed differences in the ratio of “new” responses during the Recognition Phase as a function of condition (target, lure, foil/distant lure) in both tasks (Object: *M_targe_*_t_ = 0.28, *SD* = 0.13, *M_lure_* = 0.43, *SD* = 0.14, *M_foil_* = 0.83, *SD* = 0.1, *F*(2, 76) = 264.99, *p* < .001, *η^2^_generalized_* = 0.78, Location: *M_targe_*_t_ = 0.41, *SD* = 0.13, *M_lure_* = 0.54, *SD* = 0.14, *M_distant_* = 0.58, *SD* = 0.11, *F*(2, 76) = 19.63, *p* < .001, *η^2^_generalized_* = 0.25). We then compared the ratio of “new” responses between conditions directly with paired sample t-tests. As a reminder, all reported *p* values of the post hoc comparisons are FDR adjusted (Benjamini & Hochberg, 1995). In the Object Task, post hoc paired sample t-tests showed a significant difference between each combination of conditions (target - lure: *t*(38) = -7.09, *p* < .001, target - foil: *t*(38) = -19.47, *p* < .001, lure - foil: *t*(38) = -16.63, *p* < .001). In the Location Task, post hoc paired sample t-tests showed a significant difference between target and lure trials (*t*(38) = -4.39, *p* < .001), and target and distant lure trials (*t*(38) = -5.59, *p* < .001), but not between lure and distant lure trials (*t*(38) = -1.69, *p* = .099). Importantly, the significant differences in the ratios of “new” responses between targets and lures, and targets and foils/distant lures in both tasks demonstrated that participants discriminated between previously seen and changed trials.

#### 3.1.2 Discriminability (d’) of conditions

Following previous work with a two-response (old/new) version of the mnemonic discrimination task (Loiotile & Courtney, 2015; Stark et al., 2015), we replicated the above results using a signal detection measure of discriminability (*d’*). A *d’* between each pair of conditions revealed that the discriminability of all combinations of conditions was significantly higher than 0 in the Object Task (lure-target: *M_lure-target_* = 0.46, *SD* = 0.4, *t*(39) = 7.00, *p* < .001, foil-lure: *M_foil-lure_* = 1.23, *SD* = 0.5, *t*(39) = 15.12, *p* < .001, foil - target: *M_foil-target_* = 1.7, *SD* = 0.63, *t*(39) = 16.47, *p* < .001). In the Location Task, it was significantly higher than 0 between lures and targets (*M_lure-target_* = 0.34, *SD* = 0.49, *t*(39) = 4.29, *p* < .001), and distant lures and targets (*M_distant-target_* = 0.47, *SD* = 0.55, *t*(39) = 5.2, *p* < .001), but not between distant lures and lures (*M_distant-lure_* = 0.12, *SD* = 0.42, *t*(39) = 1.81, *p* = .078).

#### 3.1.3 The effect of trial similarity on the probability of a “new” response

In the above analyses, we assigned the types target, lure and foil/distant lure based on the similarity between the recognition trial and the first encoding trial in which the fractal was presented. However, as each fractal was presented twice during encoding (either in an exactly repeated or similar version, see Fig. 2a and Fig. 3a), we hypothesized that the similarity between the recognition trial and the *second* presentation of the trial will also modulate participants’ performance. In addition, the ANOVAs presented above disregard trial-level similarity information. Therefore, in a follow-up analysis, we calculated the item and spatial similarity between each recognition trial and its first and second encoding presentation, and we assessed the relationship between each similarity measure and the probability of a “new” response using binomial generalized linear mixed effects modeling with random intercepts (see Methods).

The probability of a “new” response in the Object Task was significantly associated with the first presentation item similarity (*p* < .001, Supplementary Table S1 - Model A) and the second presentation item similarity (*p* < .001, Fig. 2c, Supplementary Table S1 - Model B). Adding both similarity measures to the model (Supplementary Table S1 - Model C) improved the fit significantly, and resulted in a slightly lower Akaike Information Criterion (AIC, Supplementary Table S2). In this model, only the effect of the similarity between the second presentation and the recognition trial remained significant (*p* < .001).

The probability of a “new” response in the Location Task was significantly associated with the first (*p* < .001, Supplementary Table S3 - Model A) and second (*p* < .001, Supplementary Table S3 - Model B) presentation’s spatial similarity (Fig. 3c). Adding both similarity measures to the model (Supplementary Table S3 - Model C) improved the fit, and resulted in a slightly lower AIC value (Supplementary Table S4). In this model, both the first presentation similarity (*p* < .001) and second presentation similarity (*p* = .006), as well as their interaction (*p* < .001), were significantly associated with the probability of a “new” response.

### 3.2 Object Encoding: Univariate ROI results

#### 3.2.1 Presentation rank by trial type model

In the Object Task, a region-wise 2-by-2 repeated measure ANOVA of presentation rank (first and second) and trial type (similar and repeat) revealed a main effect of presentation rank in the following regions: left SUB (*F*(1, 38) = 7.91, *p* = .023, *η^2^_partial_* = 0.17), left PRC (*F*(1, 38) = 16.07, *p* = .002, *η^2^_partial_* = 0.3), and right PRC (*F*(1, 38) = 25.35, *p* < .001, *η^2^_partial_* = 0.4). The effect in the left CA3DG was not significant after adjusting for the FDR (left CA3DG (*F*(1, 38) = 5.79, *p* = .053, *p_unadjusted_* = .021, *η^2^_partial_* = 0.13). The main effect of presentation rank was due to the lower activity level at Presentation 2 than Presentation 1 in all regions. We did not find a main effect of trial type (all *p’*s > .07), or any significant difference between first presentation of similar trials and first presentation of repeat trials in any area (all *p’*s > .2). Notably, we did not find any significant effect in either the left or right PHC (all *p’*s > .1*),* indicating that the experimental manipulations in the Object Task left the PHC largely unaffected. Based on our a priori hypothesis that the CA3DG and PRC are involved in object pattern separation, which should result in higher activity to similar trials than repeats, we directly compared the similar and repeat trials with paired sample t-tests in these areas. The difference between similar trials and repeats was significant in both the left PRC (*M*_similar_ = 0.05, *SD*_similar_ = 0.13, *M*_repeat_ = 0.02, *SD*_repeat_ = 0.14, *t*(38) = 2.51, *p* = .028) and right PRC (*M*_similar_ = 0.07, *SD*_similar_ = 0.16, *M*_repeat_ = 0.03, *SD*_repeat_ = 0.16, *t*(38) = 2.34, *p* = .037), suggesting that the PRC was sensitive to the object similarity manipulation. However, the difference between similar trials and repeats was not significant in either the left (*M*_similar_ = 0.02, *SD*_similar_ = 0.16, *M*_repeat_ = 0.0, *SD*_repeat_ = 0.17, *t*(38) = 1.23, *p* = .271) or right CA3DG (*M*_similar_ = 0.06, *SD*_similar_ = 0.17, *M*_repeat_ = 0.04, *SD*_repeat_ = 0.16, *t*(38) = 1.55, *p* = .257). We observed a strong repetition suppression to repeats relative to first presentation of repeats bilaterally in the PRC (left PRC: *M*_first_rep_ = 0.07, *SD*_first_rep_ = 0.14, *M*_repeat_ = 0.02, *SD*_repeat_ = 0.14, *t*(38) = 3.26, *p* = .007; right PRC: *M*_first_rep_ = 0.1, *SD*_first_rep_ = 0.16, *M*_repeat_ = 0.03, *SD*_repeat_ = 0.16, *t*(38) = 3.8, *p* = .002). This comparison did not reveal a significant difference in the CA3DG (left CA3DG: *M*_first_rep_ = 0.04, *SD*_first_rep_ = 0.16, *M*_repeat_ = 0.0, *SD*_repeat_ = 0.17, *t*(38) = 1.75, *p_unadjusted_* = .089; right CA3DG: *M*_first_rep_ = 0.06, *SD*_first_rep_ = 0.16, *M*_repeat_ = 0.04, *SD*_repeat_ = 0.16, *t*(38) = 1.78, *p_unadjusted_* = .083).

To directly test whether the PRC was more sensitive to object information in our task than the CA3DG, we ran a repeated measures ANOVA with the within factors presentation rank (first vs. second), trial type (similar vs. repeat), area (PRC vs. CA3DG), and hemisphere. Comparing the PRC and the CA3DG revealed a main effect of presentation rank (*F*(1, 38) = 18.8, *p* < .001), an interaction between presentation rank and area (*F*(1, 38) = 5.3, *p* = .027), and a three-way interaction between presentation rank, area, and hemisphere (*F*(1, 38) = 6.29, *p* = .017). The interaction effect between presentation rank and area expresses that there was a stronger decrease in activity for second presentations (repeats and similar trials) in the PRC than in the CA3DG.

#### 3.2.2 Similarity level model

**Fig. 5.**
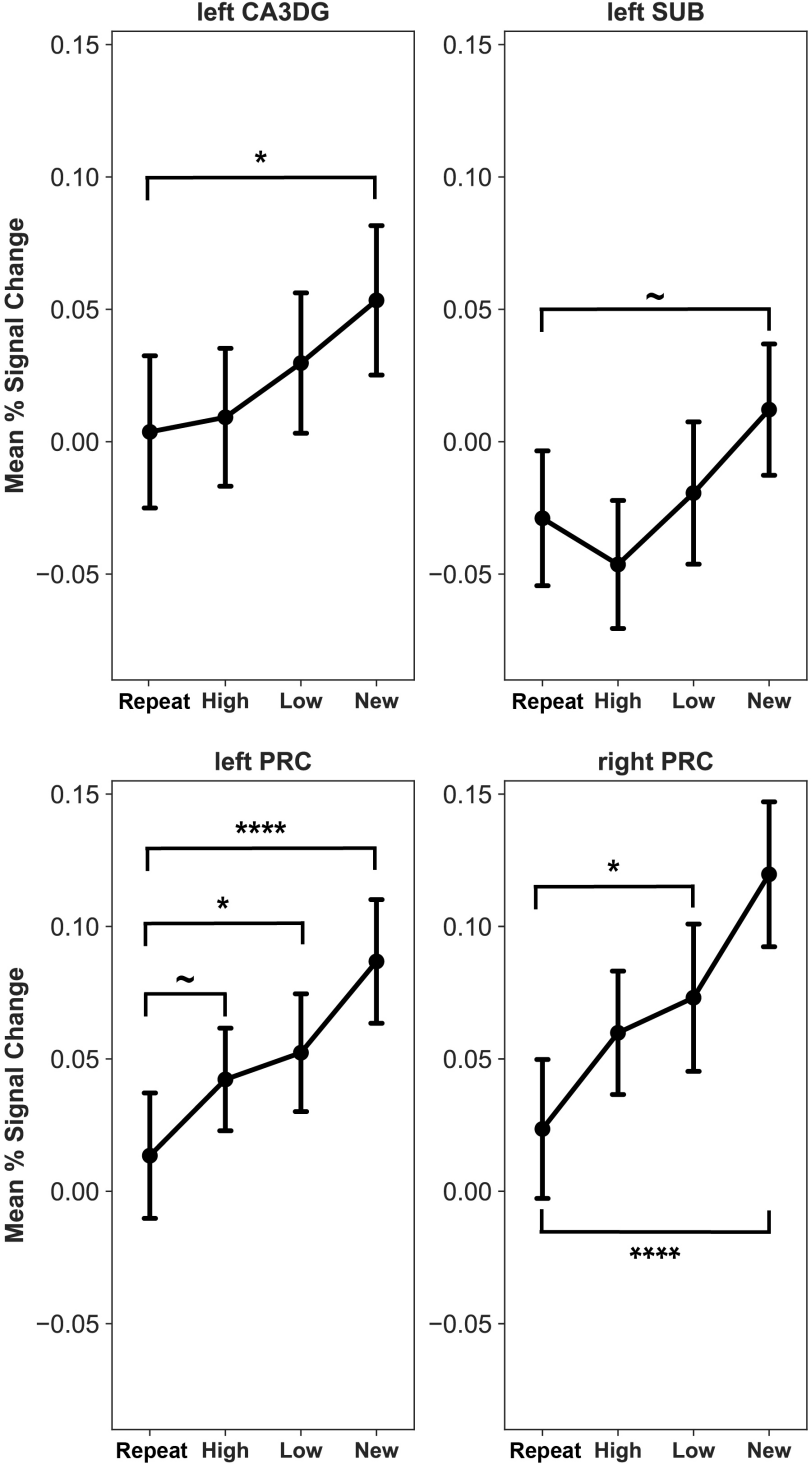
Similarity-dependent activations during Object Encoding. The PRC expresses a significant increase to low similarity object trials bilaterally. Significant differences relative to repeats (unadjusted) are denoted with a symbol: ~*p* < .06, *p < .05, ****p < .0001. Responses from all areas that showed a significant effect in the previous 2-by-2 model were included in the figure. Abbreviations: CA3DG—cornu Ammonis 3 and dentate gyrus, SUB—subiculum, PRC—perirhinal cortex. Error bars represent the standard error of mean.

As a follow-up, we binned similar trials in the encoding into high- and low-similarity trials, which enabled us to analyze percent signal change as a function of similarity. For this analysis, we merged all first presentations into one explanatory variable, given that previously we did not find a baseline difference between the first presentations of different types. This resulted in four regressors of interest: Repeat, High-Similarity, Low-Similarity, and New trials. Item similarity level-dependent activations from each region that showed a main effect of presentation rank in the previous balanced model were tested with a one-way repeated measures ANOVA. Object similarity level-dependent activations from each of these regions are shown in Figure 5. We found a significant effect of similarity in each region included in this analysis: left CA3DG (*F*(3, 114) = 3.35, *p* = .022, *η^2^_partial_* = .08), left SUB (*F*(3, 114) = 3.96, *p* = .01, *η^2^_partial_* = .09), left PRC (*F*(3, 114) = 9, *p* < .001, *η^2^_partial_* = .19), right PRC (*F*(3, 114) = 12.04, *p* < .001, *η^2^_partial_* = .24). Post hoc comparisons revealed that low similarity trials elicited significantly higher activation than repeats in the bilateral PRC (left PRC: *M_low_sim_* = 0.05, *SD_low_sim_* = 0.14, *M_repeat_* = 0.01, *SD_repeat_* = 0.15, *t*(38) = 2.43, *p* = .03, right PRC: *M_low_sim_* = 0.07, *SD_low_sim_* = 0.17, *M_repeat_* = 0.02, *SD_repeat_* = 0.16, *t*(38) = 3.2, *p* = .004). We also noted an increase to high similarity trials in the left PRC; however, this difference was not significant (*M_high_sim_* = 0.04, *SD_high_sim_* = 0.12, *t*(38) = 1.96, *p* = .068, *p_unadjusted_* = .057). No other areas expressed significant sensitivity to similar trials relative to repeats.

#### 3.2.3 Brain–behavioral correlations in the Object Task

**Fig. 6.**
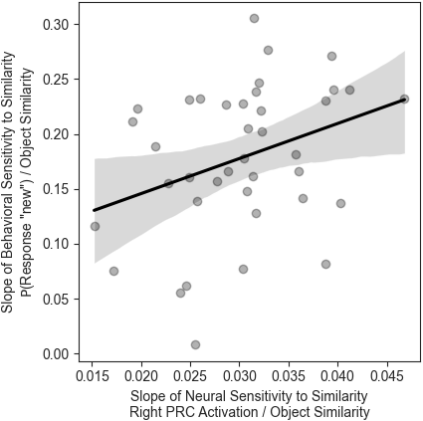
The association between the right PRC signal and mnemonic discrimination in the object domain. The behavioral sensitivity to object similarity—operationalized as the individual slope of similarity dependent “new” responses—correlates with the steepness of the right PRC’s response to the level of object similarity.

To assess whether medial temporal lobe signals were related to participants’ item discrimination performance, we first obtained the participant-specific slopes of sensitivity to item similarity from each region that showed any modulation by the Object Encoding conditions (left CA3DG, left SUB, left and right PRC). We achieved this by building linear mixed effects models, predicting each region’s activity based on the level of similarity (Repeat, High Similarity, Low Similarity, New) with random slopes and random intercepts, and then extracted the participant-specific coefficients. Likewise, we used participant-specific slopes of behavioral sensitivity to item similarity, obtained from linear mixed models predicting “new” responses based on the two similarity variables (between the first encoding presentation and recognition, and the second encoding presentation and recognition). We used Spearman correlations to assess the relationship between the neural and behavioral measures of item similarity sensitivity, and controlled for the FDR as before. Sensitivity of the right PRC correlated significantly with participants’ behavioral sensitivity to the similarity between the second encoding trial and the recognition trial (*r* = 0.41, *p* = .035; Fig. 6), indicating that the steeper the response of the right PRC was to increasing levels of similarity, the higher the behavioral sensitivity the participant expressed to the similarity manipulation, which manifested as the probability of giving a “new” response to lure and foil fractals. Additionally, we found a marginally significant negative correlation between the sensitivity of the left CA3DG to object similarity and behavioral sensitivity to the second similarity measure, but this association was not significant after adjusting for multiple comparisons (*r* = -0.31, *p* = .1, *p_unadjusted_* = .051). No other ROI revealed any significant correlation between behavioral and neural sensitivity to similarity (all *r’*s < |.2|, *p’*s > .1).

### 3.3 Location Encoding: Univariate ROI results

#### 3.3.1 Presentation rank by trial type model

In the Location Task, a region-wise 2-by-2 repeated measure ANOVA of presentation rank (first and second) and trial type (similar and repeat) revealed a main effect of presentation rank in the following regions: left CA3DG (*F*(1, 38) = 8.53, *p* = .012, *η^2^_partial_* = .18), right CA3DG (*F*(1, 38) = 11.01, *p* = .008, *η^2^_partial_* = .22), right SUB (*F*(1, 38) = 10.85, *p* = .008, *η^2^_partial_* = 0.22), left PHC (*F*(1, 38) = 9.71, *p* = .009, *η^2^_partial_* = 0.2), and right PHC (*F*(1, 38) = 10.47, *p* = .008, *η^2^_partial_* = 0.22). The effect in the left CA1 was not significant after correcting for multiple comparisons (*F*(1, 38) = 4.31, *p* = .074, *p_unadjusted_* = .045, *η^2^_partial_* = .1). The main effect of presentation rank was driven by a lower signal at Presentation 2. In contrast, we found no effect in either the left or the right PRC (both *p’*s > .1*)*, suggesting that this area did not show repetition suppression during Location Encoding. We did not find a main effect of trial type (all *p’*s > .2), or any significant difference between first presentation of similar trials and first presentation of repeat trials in any area (all *p’*s > .2), and thus we concluded that there was no baseline difference between the first presentations of different types. Post hoc paired sample t-test in the left PHC revealed that while activity was significantly higher for first presentations of later repeats than repeats (*M*_first_rep_ = 0.06, *SD*_first_rep_ = 0.14, *M*_repeat_ = 0.01, *SD*_repeat_ = 0.1, *t*(38) = 2.8, *p* = .024), there was no significant difference between first presentations of later similar trials and similar trials (M_first_sim_ = 0.05, *SD*_first_sim_ = 0.12, *M*_similar_ = 0.04, *SD*_similar_ = 0.12, *t*(38) = 1.31, *p* = .236). This pattern was also found in the right PHC (first presentation of later repeats vs. repeats: *M*_first_rep_ = 0.09, *SD*_first_rep_ = 0.14, *M*_repeat_ = 0.04, *SD*_repeat_ = 0.11, *t*(38) = 2.87, *p* = .022, first presentations of later similar trials vs. similar trials: M_first_sim_ = 0.08, *SD*_first_sim_ = 0.13, *M*_similar_ = 0.06, *SD*_similar_ = 0.12, *t*(38) = 1.64, *p* = .163). Activity was additionally significantly higher for similar trials than repeats in the left PHC, but this effect did not persist after adjusting for multiple comparisons (*t*(38) = 2.22, *p* = .065, *p_unadjusted_* = .033). We expected that the activation to similar trials would be higher than to repeats in the CA3DG, given its hypothesized role in pattern separation; however, the difference was not significant in either the left (*M*_similar_ = 0.03, *SD*_similar_ = 0.14, *M*_repeat_ = 0.05, *SD*_repeat_ = 0.14, *t*(38) = -1.17, *p* = .301) or right CA3DG (*M*_similar_ = 0.04, *SD*_similar_ = 0.15, *M*_repeat_ = 0.03, *SD*_repeat_ = 0.14, *t*(38) = 0.4, *p* = .69). We found repetition suppression to repeats relative to the first presentation of repeats in the right CA3DG, which was not significant after adjusting for the FDR (*M*_first_rep_ = 0.06, *SD*_first_rep_ = 0.15, *M*_repeat_ = 0.03, *SD*_repeat_ = 0.14, *t*(38) = 2.06, *p* = .092, *p_unadjusted_* = .046). Note, however, that the main effect of presentation rank in the bilateral CA3DG nonetheless indicates that the activity to similar trials and repeats was lower than to first presentations in this region.

To directly test whether the PHC was more sensitive to the location information in our task than CA3DG, we ran a repeated measures ANOVA with the within factors presentation rank (First vs. Second), trial type (Similar vs. Repeat), area (PHC vs. CA3DG), and hemisphere. Comparing the PHC and the CA3DG revealed a main effect of presentation rank (*F*(1, 38) = 18.7, *p* < .001) and a three-way interaction between presentation rank, trial type, and area (*F*(1, 38) = 5.51, *p* = .024).

#### 3.3.2 Similarity level model

**Fig. 7.**
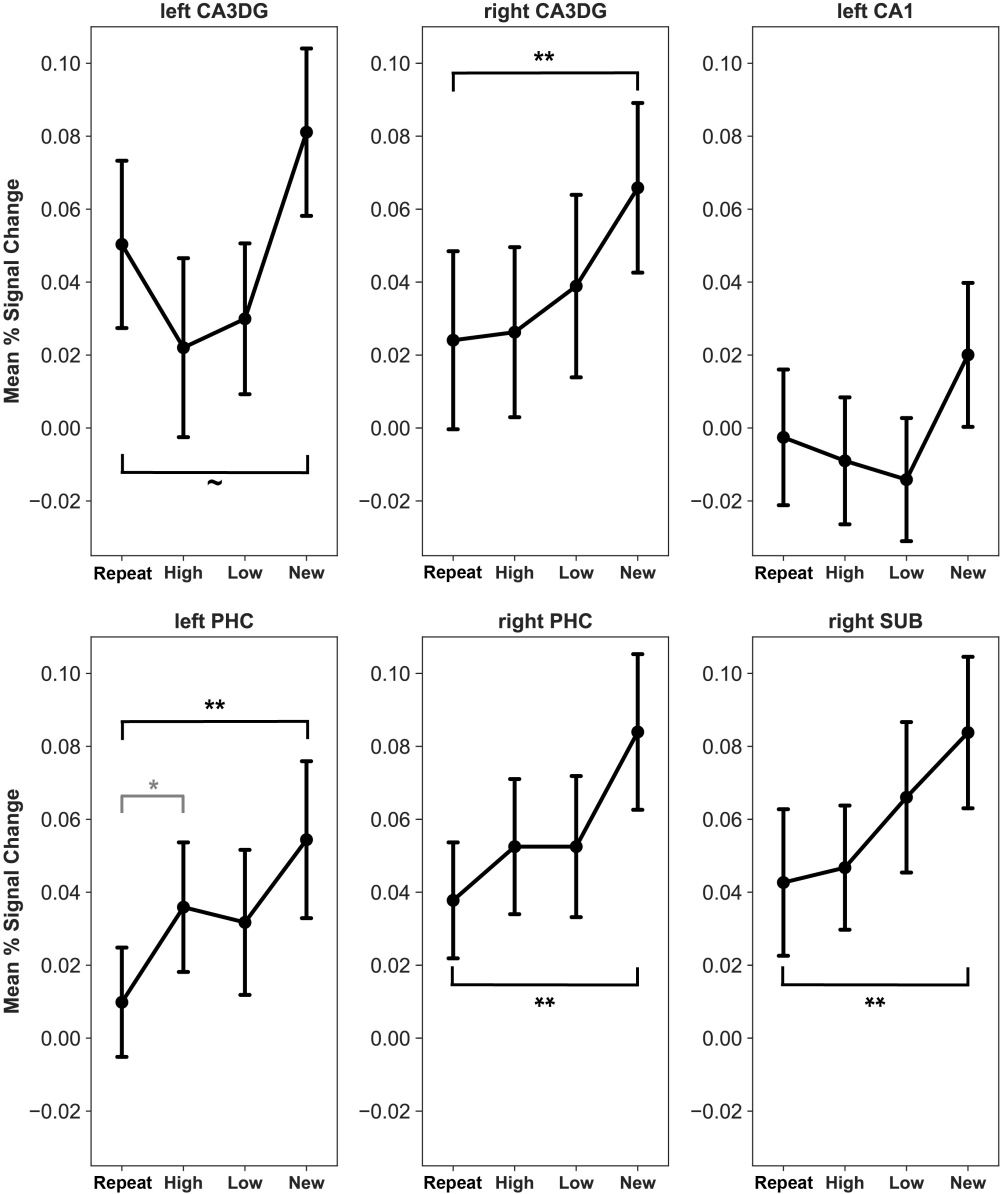
Similarity-dependent activations during Location Encoding. Responses from all areas that showed a significant effect in the previous 2-by-2 model were included in the figure. Significant differences relative to repeats (unadjusted) are denoted with a symbol: ~*p* < .06, *p < .05, **p < .01. The comparison between the repeat and high similarity trials in the left PHC (lower left panel, marked with gray color) was not significant after correcting for multiple comparisons. Abbreviations: CA1—cornu Ammonis 1 and 2, CA3DG—cornu Ammonis 3 and dentate gyrus, SUB—subiculum, PHC—parahippocampal cortex. Error bars represent the standard error of mean.

We binned similar trials of the Location Encoding into high- and low-similarity trials to analyze percent signal change as a function of spatial similarity. Again, all first presentation trials were merged into one regressor, creating four similarity regressors: Repeat, High-Similarity, Low-Similarity, and New trials. In the statistical analysis of the extracted percent signal change values, we included all regions of interest that showed a response to the task manipulation in the previous analysis, that is left CA1, left and right CA3DG, right SUB, left PHC, and right PHC. Spatial similarity level-dependent activations from each of these regions are shown in Figure 7. We assessed the sensitivity to spatial similarity in a one-way repeated measures ANOVA. We found a main effect of similarity in all regions included in this analysis (left CA3DG: *F*(3, 114) = 4.24, *p* = .007, *η^2^_partial_* = .1, right CA3DG: *F*(3, 114) = 2.98, *p* = .034, *η^2^_partial_* = .07, right SUB: *F*(3, 114) = 3.17, *p* = .033, *η^2^_partial_* = .07, left PHC: *F*(3, 114) = 3.98, *p* = .01, *η^2^_partial_* = .09, right PHC: *F*(3, 114) = 2.93, *p* = .037, *η^2^_partial_* = .07), except the left CA1 (F(3, 114) = 2.17, *p* = .094, *η^2^_partial_* = .05). Post hoc comparisons revealed that the left PHC was the only area where the difference between repeated and highly similar trials was significant, albeit this difference did not persist after adjusting for multiple comparisons (*M_high_sim_* = 0.04, *SD_high_sim_* = 0.11, *M_repeat_* = 0.01, *SD_repeat_* = 0.1, *t*(39) = 2.19, *p* = .104, *p_unadjusted_* = .035).

#### 3.3.3 Brain behavioral correlations in the Location Task

We then assessed the associations between the neural responses of the medial temporal ROIs and mnemonic discrimination in the spatial domain. For this purpose, we obtained the participant-specific slopes of sensitivity to spatial similarity from linear mixed effects models that predicted each region’s responses based on the level of similarity. All regions that exhibited some modulation by the Location Encoding conditions were included in this analysis (left CA1, left and right CA3DG, right SUB, left PHC, and right PHC). Each participant’s behavioral sensitivity (i.e. individual slope) to the spatial similarity between the first encoding presentation and the recognition trial, and the second encoding presentation and the recognition trial was used as a behavioral outcome. We ran Spearman correlations, and found an association between the right CA3DG response and the behavioral sensitivity to the similarity between the second encoding trial and the recognition trial (*r* = 0.34, *p_unadjusted_* = .034, Supplementary Fig. S1) that did not remain significant after adjusting for the FDR (*p* = .168).

### 3.4 CA3DG responses combined across tasks

The results above indicated that the PRC and PHC showed more pronounced responses than the CA3DG during the Object and Location Encoding, respectively, and that the CA3DG showed little sensitivity to similar trials. As a follow-up analysis, we combined the extracted percent signal change values of the CA3DG from the two tasks, and conducted one repeated measures ANOVA per hemisphere with similarity level (repeat, high similarity, low similarity, new) and task (Object versus Location) as within-subject factors. In both the left and right CA3DG, we found a main effect of similarity level (left CA3DG: *F*(3, 114) = 8.18, *p* < .001, right CA3DG: *F*(3, 114) = 6.13, *p* < .001), but no effect of task and no task–similarity interaction (all *p’*s > 0.3). In the left CA3DG post hoc paired sample t-tests between similarity levels revealed a significant difference between repeat and new trials (*t*(77) = 3.32, *p* = .004), high similarity trials and new trails (*t*(77) = 4.22, *p* < .001), and low similarity trials and new trials (*t*(77) = 3.07, *p* < .006). There was no significant difference between either high or low similarity and repeat trials (all *p’*s > 0.3). In the right CA3DG post hoc paired sample t-tests between similarity levels revealed a significant difference between repeat and new trials (*t*(77) = 3.95, *p* = .001), and high similarity trials and new trails (*t*(77) = 3.16, *p* = .007). There was no significant difference between high similarity and repeat trials (*p* > 0.5). The difference between the low similarity and repeat trials was marginally significant without adjustment (*t*(77) = 1.91, *p* = .118, *p_unadjusted_* = .059). Further, low similarity trials did not significantly differ from new trials (*t*(77) = 1.08, *p_unadjusted_* = .285). Altogether, these results are in line with the previous task-wise analysis, that is, that the CA3DG shows repetition suppression to second presentation trials, and although there seems to be some similarity-based modulation in the CA3DG, the difference between repeated and similar trials is not statistically significant.

### 3.5 Task-dependent responses in the parahippocampal and perirhinal cortex

**Fig. 8.**
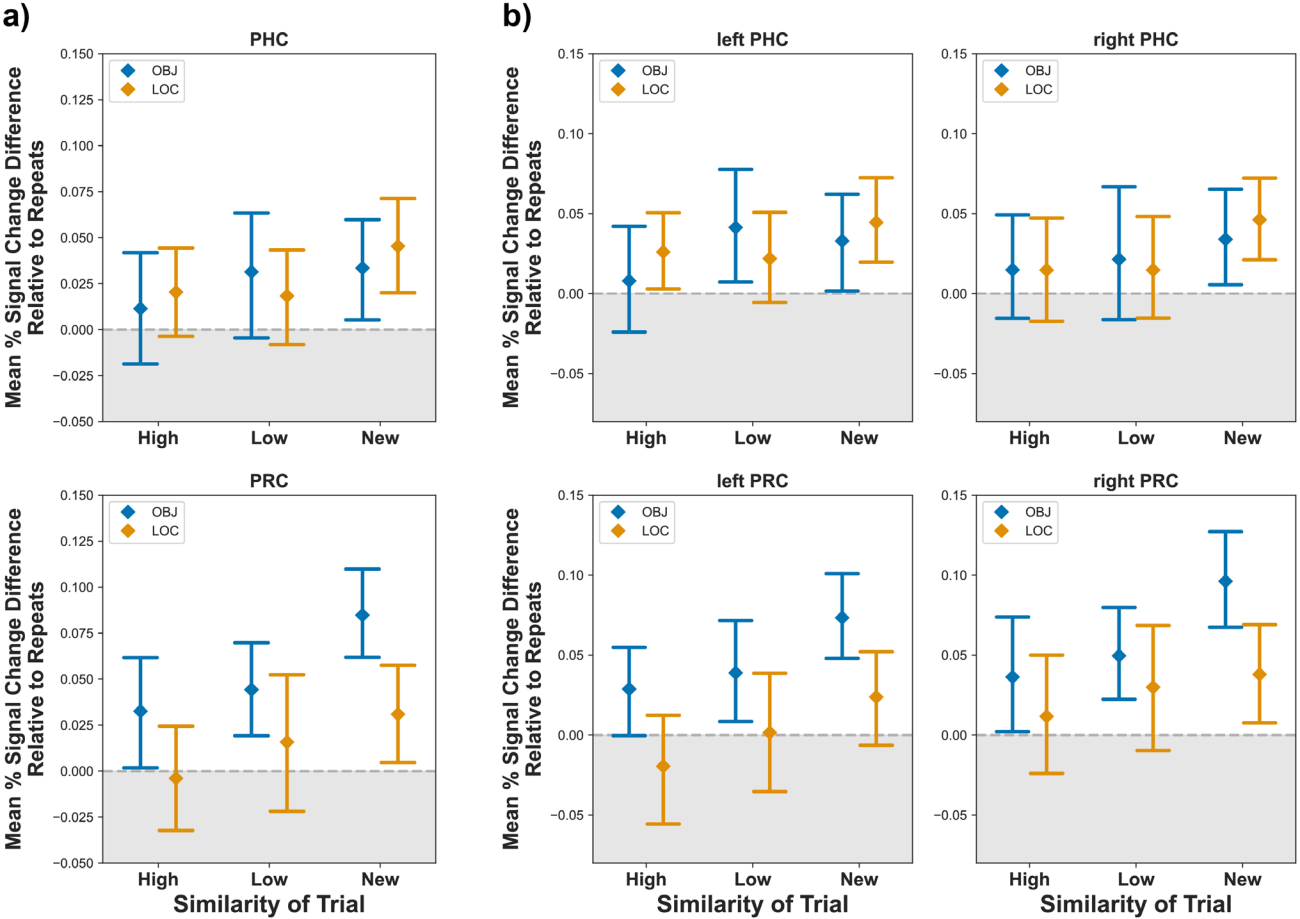
Similarity level-dependent responses of the parahippocampal and perirhinal cortices across the two tasks. Responses to repeats were extracted from the mean percent signal change values of each similarity level for visualization purposes. Values around and below 0, therefore, can be considered repetition suppression. Panel (a) shows the cross-hemisphere average and panel (b) shows the signal from each hemisphere. A three-way interaction between similarity level, task and area indicates that similarity-dependent responses differ based on task context. Error bars represent 95% confidence intervals.

We tested whether the activity of PRC and PHC statistically differed depending on the task context, that is, whether the PRC is more engaged in object interference resolution than the PHC and vice versa. To this end, we merged data from the two tasks and two regions, and conducted a repeated measures ANOVA with area (PHC versus PRC), hemisphere, similarity level (Repeat, High Similarity, Low Similarity, New), and task (Object versus Location) as within-subject factors. We found a significant main effect of similarity level (*F*(3, 114) = 13.07, *p* < .001), and a main effect of hemisphere (*F*(1, 38) = 13.07, *p* = .043). The main effect of hemisphere was due to higher mean percent signal change in the right hemisphere (*t*(623) = 4.08, *p* < .001). We did not find a main effect of either task (*F*(1, 38) = 0.01, *p* = .922) or area (*F*(1, 38) = 1.28, *p* = .266). Importantly, the analysis revealed a significant three-way interaction between similarity level, area, and task (*F*(3, 114) = 3.87, *p* = .011). This interaction indicates that depending on the task context—and thus the type of information that needed to be attended—similarity-induced interference differentially modulated the activity of the PRC and PHC (Fig. 8).

### 3.6 Whole-brain results

#### 3.6.1 Univariate activations

**Fig. 9.**
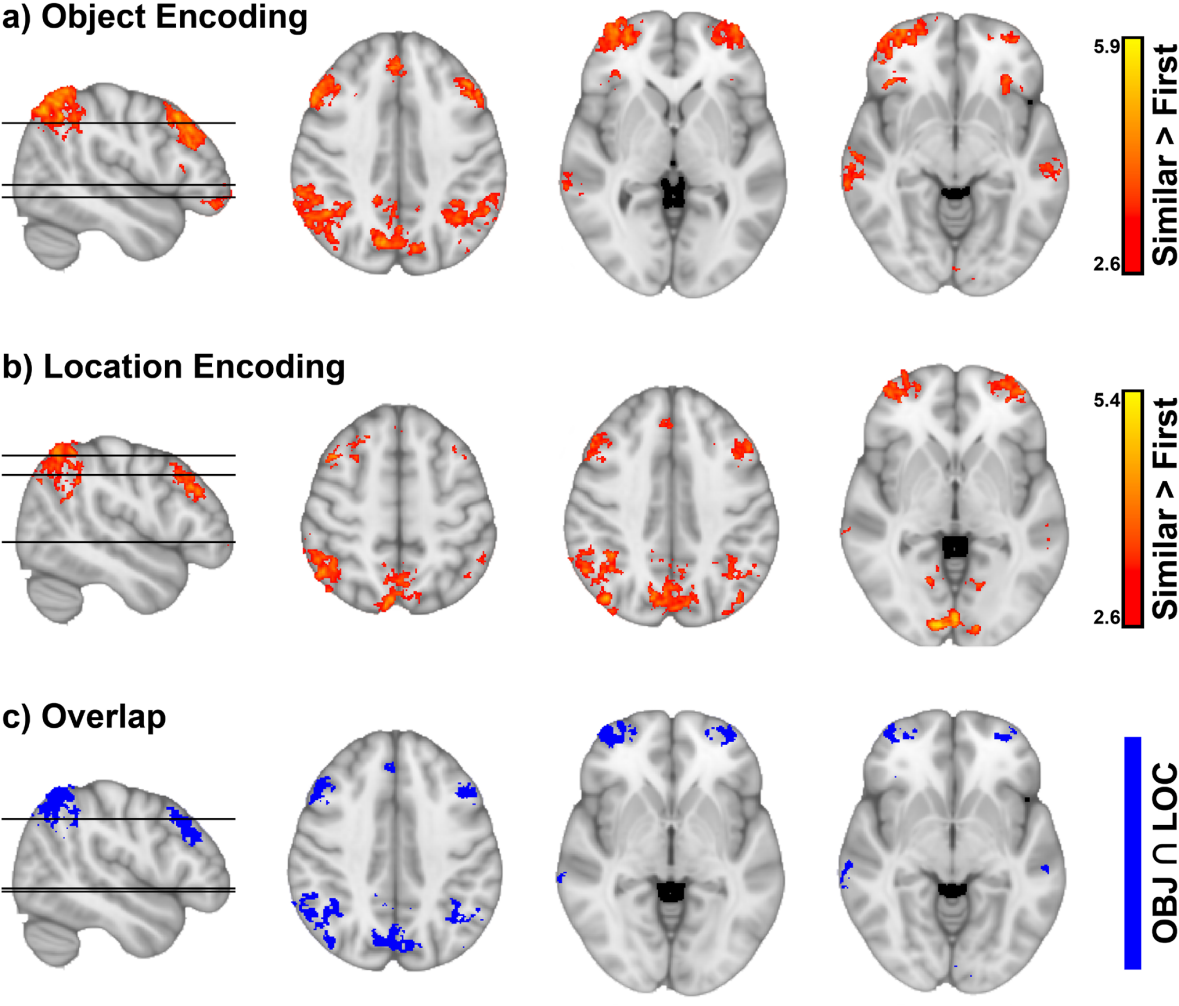
Clusters of significant activation to similar > first trials. Whole brain activations to similar > first presentation trials showed an overlap between the Object and Location Tasks. Frontoparietal regions were activated by both task contexts. (a) Significant clusters of activation during the Object Encoding. (b) Significant clusters of activation during the Location Encoding. (c) Overlap of significant clusters in the Location and Object Tasks.

**Fig. 10.**
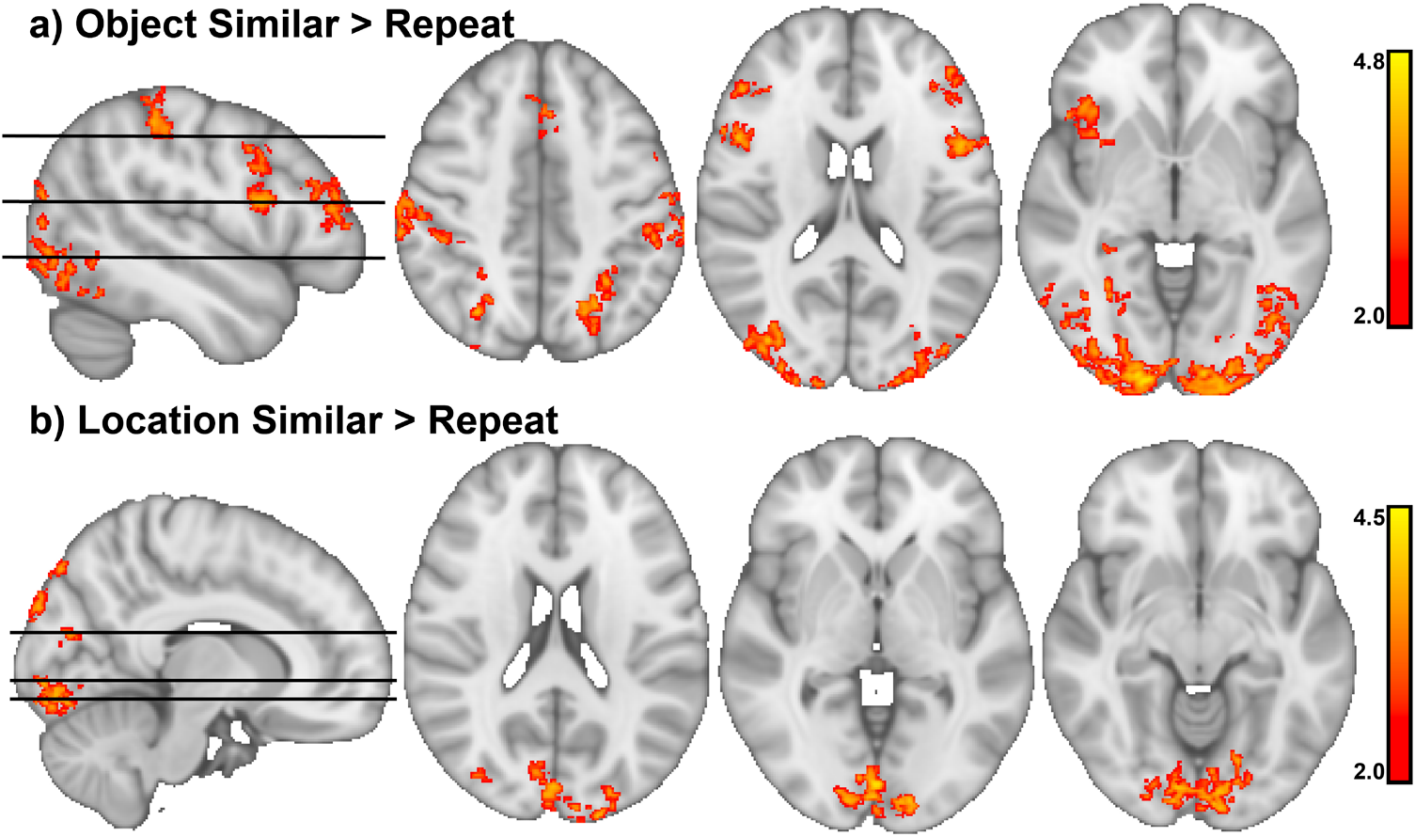
Clusters of significant difference between similar and repeat trials. The figure depicts the contrast (similar > first presentation) > (repeat > first presentation), which isolates regions specifically activated during the encoding of similar stimuli. (a) During similar object trials, occipital areas, as well as the superior parietal gyrus, the inferior frontal gyrus, and middle frontal gyrus, were activated more than during repeat trials. (b) During similar location trials, occipital areas were activated more so than during repeat trials.

We found 13 clusters in the Object Encoding (Fig. 9a) and 12 clusters in the Location Encoding (Fig. 9b) that showed significantly higher activity to similar trials than their first presentation, summarized in Supplementary Tables S6 and S7. Importantly, the clusters derived from the Location and Object Tasks moderately overlapped (Dice–Sørensen coefficient = 0.54, Fig. 9c), and both covered regions belonging to the frontoparietal control network, that is, the middle frontal gyrus and the supramarginal gyrus (Dixon et al., 2018; Uddin et al., 2019) and the angular gyrus. No significant clusters were found in the medial temporal lobe. Results from the contrast (*similar - first presentation of later similar trials)* *>* *(repeats - first presentation of later repeats)* are shown in Figure 10 and reported in Supplementary Tables S4 and S5. Untresholded z maps were uploaded to Neurovault (Gorgolewski et al., 2016) and analyzed with Neurosynth (Yarkoni et al., 2011) to obtain the most closely associated neurocognitive terms based on the meta-analysis of the available fMRI data. This analysis supported our interpretation that frontoparietal regions were activated by similar trials. In turn, the terms “default mode”, “memory retrieval”, “recognition”, and “episodic memory” were less associated with the similar > first activation maps. Results of the Neurosynth analysis (including the top 10 associated terms) for the similar > first contrast are reported in the Supplementary Tables S8–S11.

#### 3.6.2 gPPI with frontal and parietal seeds

We report results from each seed region’s interaction with the similar (similar PPI contrast) and repeat condition (repeat PPI contrast), which reflect increases in connectivity relative to the baseline. Cluster statistics for the similar PPI contrast is given in Table 1 (Object Task) and Table 2 (Location Task). Additionally, we report results from the contrast similar PPI > repeat PPI, which indicate if the seed region interacts with some areas more in the similar condition than the repeat condition. One gPPI analysis was run per ROI and task. In the Object Task, only the right angular gyrus and the right middle frontal cortex seeds expressed increased functional connectivity with other regions. We found 1 cluster (256 voxels, max. *z* = 3.42, *p* = .044) of increased connectivity during the encoding of similar object trials between the right angular gyrus seed and the left supramarginal gyrus and left angular gyrus. The right middle frontal gyrus seed increased its connectivity with two clusters: one located in the left angular cortex (301 voxels, *z* = 3.5, *p* = .011), and the other in the posterior region of the middle temporal gyrus (343 voxels, *z* = 3.64, *p* = .004). No significant clusters were found for the contrast similar PPI > repeat PPI in the Object Task. This indicates that the interaction between similar trials and functional connectivity did not statistically differ from the interaction between repeat trials and functional connectivity. Note, however, that no significant clusters were found for the repeat PPI contrast in the Object Task.

**Table 1.** Significant clusters in the Object Task for the similar PPI contrast.

Seed region	Areas in cluster	N. voxels	Max. *z*	Cluster *p*
Right angular gyrus	Left supramarginal gyrus, left angular gyrus	256	3.42	.044
Right middle frontal gyrus	Left angular gyrus	301	3.5	.011
	Left middle temporal gyrus	343	3.63	.004

**Table 2.** Significant clusters in the Location Task for the similar PPI contrast.

Seed region	Areas in cluster	N. voxels	Max. *z*	Cluster *p*
Left angular gyrus	Right angular gyrus and supramarginal gyrus	383	3.33	.002
	Right frontal pole	373	2.92	.002
	Right postcentral gyrus and supramarginal gyrus	282	3.05	.023
	Right supramarginal gyrus	266	3.82	.037
Right angular gyrus	Left angular gyrus, left supramarginal gyrus	256	3.42	.044
Left supramarginal gyrus	Right angular gyrus	2055	3.69	< .001
	Right frontal pole	1141	3.62	< .001
	Right middle temporal gyrus, posterior division	931	3.91	< .001
	Right posterior cingulate and precuneus	899	3.3	< .001
	Left lateral occipital cortex	426	3.44	< .001
	Left precuneus	296	3.3	.013
	Right superior frontal gyrus and frontal pole	259	3.55	.037
Right supramarginal gyrus	Right angular gyrus and lateral occipital cortex	3016	3.68	< .001
	Left frontal pole	1607	3.56	< .001
	Right middle temporal gyrus, posterior division	1455	3.47	< .001
	Bilateral lingual gyrus	1300	3.81	< .001
	Right frontal pole	1087	3.99	< .001
	Left inferior and middle frontal gyrus	1056	3.72	< .001
	Right middle frontal gyrus	845	4	< .001
	Left middle frontal gyrus	589	4.14	< .001
	Right middle frontal gyrus, precentral gyrus	549	3.1	< .001
	Bilateral precuneus	476	3.2	< .001
	Right fusiform gyrus	426	3.37	< .001
	Bilateral paracingulate gyrus	413	3.55	< .001
	Right superior frontal gyrus	405	3.33	< .001
	Bilateral posterior cingulate gyrus	402	3.43	< .001
	Left lingual gyrus, left fusiform gyrus	372	3.54	.002
	Left paracingulate gyrus, left anterior cingulate gyrus	355	3.39	.002
	Left lateral occipital cortex, left angular gyrus	350	3.59	.003
	Left orbitofrontal cortex	331	3.44	.005
	Right lateral occipital cortex	315	3.36	.007
	Right middle temporal gyrus, anterior division	304	3.13	.01
Left middle frontal gyrus	Right frontal pole	467	3.09	< .001
	Right angular gyrus, right supramarginal gyrus	328	3.41	.005

In the Location Task, we found enhanced functional connectivity between the left angular gyrus seed and the right frontal pole, right supramarginal gyrus, right postcentral gyrus, and right angular gyrus during the encoding of similar spatial trials. The left supramarginal gyrus increased its connectivity with the right frontal pole and superior frontal gyrus, right middle temporal gyrus, bilateral cingulate and precuneus cortex, right angular and supramarginal gyrus, and left lateral occipital cortex. The right supramarginal gyrus expressed a similar, but even more extensive pattern of connectivity increase, involving some additional sensory areas, such as the lingual gyrus and occipital fusiform cortex, in addition to bilateral frontal and parietal regions. As for the left middle frontal gyrus seed, only two significant clusters of connectivity increase were found, one in the right frontal pole and one in the right angular gyrus. No significant connectivity increase was found for the right angular gyrus and right middle frontal gyrus seeds for the similar PPI contrast.

For the repeat PPI contrast in the Location Task, we found six significant clusters of activations, which were anatomically consistent with but much less extensive than for the similar PPI contrast (Table 3).

**Table 3.** Significant clusters in the Location Task for the repeat PPI contrast.

Seed region	Areas in cluster	N. voxels	Max. *z*	Cluster *p*
Left angular gyrus	Right angular gyrus and supramarginal gyrus	300	3.27	.011
Left middle frontal gyrus	Right inferior frontal gyrus, right orbitofrontal cortex	718	3.2	< .001
	Right frontal pole	488	3.32	< .001
	Right precentral gyrus	366	3.05	.002
	Left postcentral gyrus, left central opercular cortex	306	3.49	.01
Left supramarginal gyrus	Left angular gyrus, left lateral occipital cortex	261	3.22	0.03

Moreover, we found two significant, anatomically overlapping clusters for the similar PPI > repeat PPI contrast in the Location Task. Both the left supramarginal gyrus (363 voxels, max. *z* = 3.79, *p* = .001) and the right middle frontal gyrus (23 voxels, max. *z* = 3.5, *p* = .006) increased their connectivity with the left occipital pole during the encoding of similar trials, and this increase was significantly higher than that in the repeat condition.

### 3.7 ROI-to-ROI functional connectivity

**Fig. 11.**
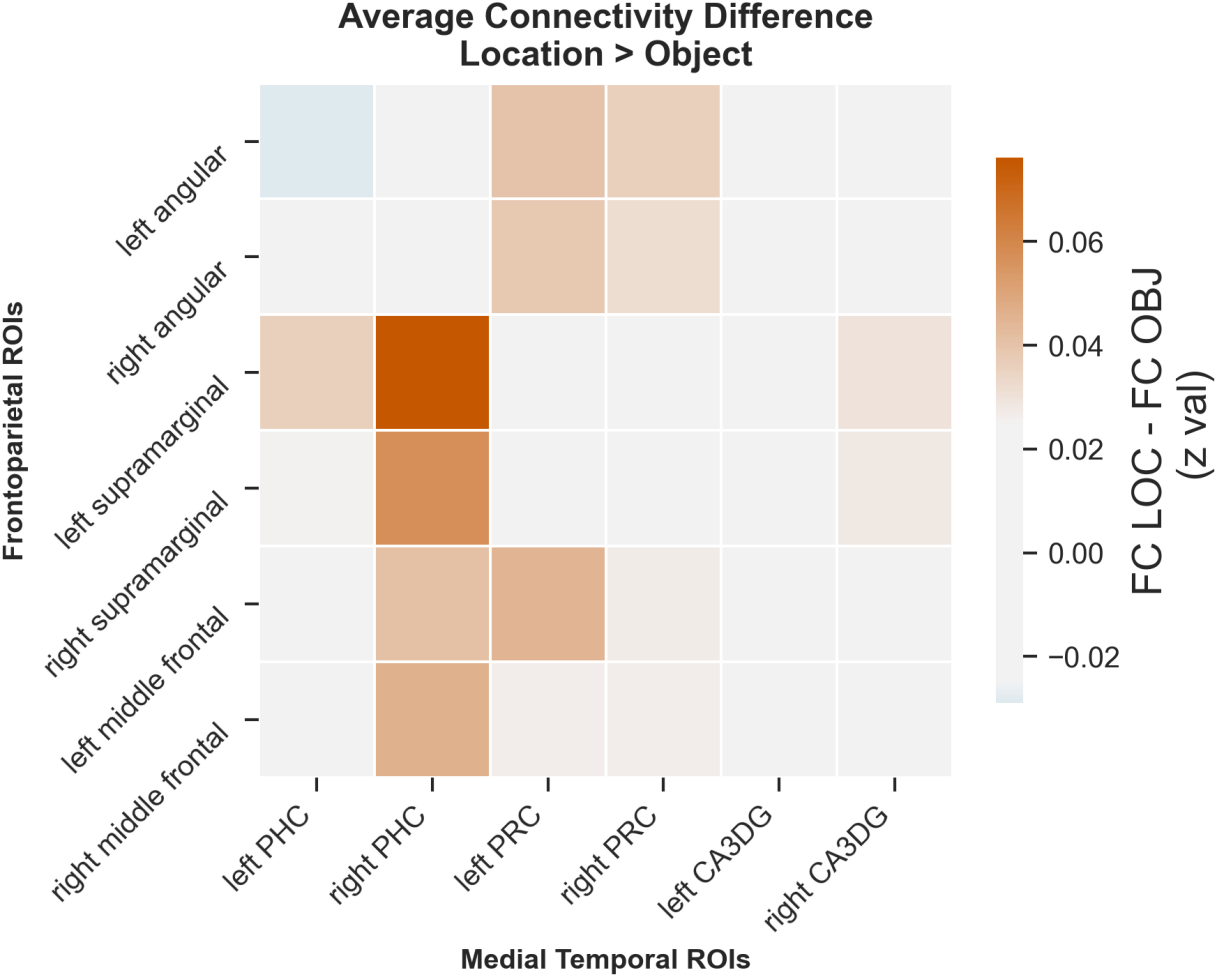
Functional connectivity differences between the Object and the Location Encoding. The connectivity between the right PHC and left supramarginal gyrus was higher during Location Encoding than during Object Encoding. The correlation between each medial temporal ROI and each frontoparietal ROI during the Object Encoding was subtracted from the Location Encoding for the purpose of visualizing the differences in connectivity between the tasks.

Each medial temporal ROI exhibited significant connectivity with the frontoparietal regions in both tasks (Supplementary Figs. S3 and S4). Further, the connectivity between the right PHC and the left supramarginal gyrus was significantly higher during the Location Encoding than the Object Encoding (*t*(39) = 2.23, *p* = .025), but this difference was not significant after adjusting for the FDR (*p* = .521). No other difference was found in the connectivity of medial temporal and frontoparietal regions between the two tasks (Fig. 11).

The associations between the behavioral variables (object and spatial similarity sensitivity) and ROI-to-ROI connectivity variables are reported in order of effect size in the supplement. Intriguingly, we found exclusively negative relationships between the connectivity of both the PHC and PRC during encoding and the behavioral sensitivity to similarity. However, none of the correlations remained significant after FDR adjustment, potentially due to the sheer number of comparisons (36 connectivity variables). We found no associations between the behavioral measures and the connectivity of the CA3DG in either task.

## 4 Discussion

Our results provide three contributions to the cognitive neuroscience of memory. First, amid the debate about the anatomical and functional independence of the two MTL pathways (Burke et al., 2018; Connor & Knierim, 2017; Lawrence et al., 2020; Nilssen et al., 2019; Rolls, 2024), our results support the notion that the perirhinal and parahippocampal cortices provide complementary information about individual items and their spatial context, respectively. Second, we demonstrate that the perirhinal and parahippocampal cortices show a sensitivity to interfering mnemonic information, even when this information is not easily verbalized and categorized. In contrast, we found only limited evidence that responses were modulated by the similarity of non-meaningful stimuli in the CA3DG. Third, our results suggest that frontoparietal regions are recruited in response to both spatial and object interference, and the connectivity of these regions increases within the network and with downstream areas during the lure condition relative to baseline. Below, we discuss the interpretation of these results, and place them within a general discussion on medial temporal lobe function and human memory.

Behaviorally, we found a significant difference between the ratio of “new” responses to target and lure trials in both the Object and Location Tasks. Moreover, we found that not only the first, but also the similarity of the second encoding trial influenced participants’ discrimination behavior during recognition. Together, these behavioral results strongly suggest that mnemonic discrimination was affected by the similarity manipulation at encoding. Therefore, we can conclude that the interference between the encoding trials provides a viable basis for assessing pattern separation at the neural level. A noteworthy difference between the Object and Location Tasks is the inclusion of foils (unique, never-before-seen items) in the Object Recognition, whereas only lures and distant lures were included in the Location Recognition. Our goal was to make the two tasks as similar as possible, but due to the difference in the type of information that was manipulated—namely, that the location information has to bind to a specific object—some asymmetry in design was unavoidable. All neuroimaging results reported here are based on the encoding phase of the tasks, and thus they are not affected by this difference during recognition.

In line with prior fMRI work (Bencze et al., 2024; Berron et al., 2018; Reagh & Yassa, 2014), our results support the view that a division of labor exists between the PRC and PHC. The PHC showed strong repetition suppression to repeated trials viewed in unchanged locations, and we found some tentative evidence that its activity was modulated by high-similarity spatial trials. Conversely, the experimental manipulations of object features elicited significant effects in the PRC, including repetition suppression to repeated trials, and activity changes as a function of similarity. A direct comparison of the effects of similarity on the PRC and PHC in the Object and Location Tasks revealed a three-way interaction of similarity, area, and task, demonstrating that the responses of these areas differed based on task context. Notably, new and repeated trials had an identical structure during Location and Object Encoding, yet the PRC showed sensitivity to the task context, and expressed stronger repetition suppression to repeated trials when the task demands matched its respective specialization. This highlights the possibility that PRC and PHC responses are modulated by top–down control processes rather than pure trial features.

The PRC and PHC showed sensitivity to high similarity trials in accordance with their task domains. In the Object Task we found that similar trials elicited significantly higher activation than repeated trials in both the left and right PRC. Moreover, the sensitivity of the PRC to object similarity was related to participants’ mnemonic discrimination behavior. Correspondingly, in the Location Task we found a tendency of high-similarity trials to elicit higher activation than repeated trials in the left PHC, albeit this result was not significant after correcting for multiple comparisons. When considered together with the result that the activation to similar trials did not differ statistically from first presentation trials in either the left or right PHC, this suggests that the PHC was sensitive to interfering location information. Although the lack of repetition suppression and the modulation of BOLD by similarity have been taken as evidence for pattern separation in the hippocampus, we refrain from qualifying such signals outside the hippocampus as pattern separation and refer to our findings as interference resolution. Future studies need to establish the mechanism of interference reduction in these regions.

In contrast to the PRC and PHC, the CA3DG did not express any significant increase in response to similar trials in either task or when responses of the two tasks were combined. Additionally, directly comparing the CA3DG with the PRC and PHC in the Object and Location Tasks revealed that the neocortical regions showed higher task sensitivity in their respective domains than the CA3DG. Specifically, we found an interaction between area (CA3DG vs. PRC) and presentation rank in the Object Task, meaning that the PRC showed a more pronounced decrease to second presentation trials than the CA3DG. In the Location Task, a three-way interaction between presentation rank (first vs. second), trial type (repeat vs. similar), and area (PHC vs. CA3DG) suggests that PHC responses were modulated more by repeats and similar trials than those of the CA3DG. These results indicate that the activity of neocortical areas in the medial temporal lobe may be sensitive to high-interference trials even when the CA3DG exhibits only moderate activity increase in response to similarity, suggesting that the hippocampus is less involved in the differentiation of non-meaningful stimuli than the neocortex. An important open question is whether interference resolution in the PRC and PHC reduces the need for pattern separation in the hippocampus, thereby diminishing its signal. Modeling activity or voxel patterns on a trial-by-trial basis and examining cross-regional relationships could provide insight. Future studies with designs optimized for single-trial modeling and multivariate analysis may test whether, and how, the interference sensitivity of upstream MTL regions contributes to the presence—or absence—of pattern separation in the CA3DG.

The behavioral analysis suggested that participants’ discrimination performance was modulated by similarity. Thus, we expected that BOLD responses during encoding would also reflect the sensitivity to object and spatial similarity, which was the case in the PRC and PHC, respectively. As for the CA3DG, we observed some similarity-related activity, which was not statistically significant (see e.g., the section “CA3DG responses combined across tasks”). Because the behavioral performance in our recognition task was below what is often observed with meaningful objects, one natural question is whether the lack of pattern separation signal in the CA3DG is due to the difficulty of the task. However, in our work, the neural activity observed during encoding is not directly reflected in the recognition performance, because each trial in the recognition phase can be related to two stimulus presentations in the encoding phase. For real-world objects, increasing the number of exemplars within a category decreases mnemonic discrimination performance (Konkle et al., 2010; Poch et al., 2019), and this is presumably the case for fractals as well. Thus, discrimination in the recognition phase was likely more difficult than during encoding. Nonetheless, we cannot exclude that reducing the encoding task difficulty by increasing the dissimilarity of fractals would enhance the similarity-dependent BOLD responses in the CA3DG. However, further increases in stimulus dissimilarity may change the nature of the task entirely, tipping the balance from lure discrimination toward foil rejection. Namely, low similarity fractal pairs were easy to discriminate perceptually (Fig. 2d), but this did not translate into a large advantage in mnemonic discrimination (Fig. 2e). However, participants showed a high foil rejection performance. Evaluating how much distortion in these abstract stimuli amounts to creating new items (foils) rather than related items is a matter of psychophysical experimentation.

Numerous previous studies suggest the dentate gyrus is involved in creating unique memories in humans (e.g., Azab et al., 2014; Bakker et al., 2008; Berron et al., 2016; Kyle et al., 2015), and some have argued for a necessary role of the dentate gyrus in mnemonic discrimination (Baker et al., 2016; Mitchnick et al., 2024). A key difference between preceding work and ours is the meaningfulness of stimuli used. Using everyday objects as stimuli may confound pattern separation and retrieval-related processes, such as pattern completion, even during encoding. Our findings with fractal stimuli may warrant re-evaluation of existing evidence on hippocampal pattern separation, and underline the need for systematic examination of its boundary conditions.

A few studies investigating pattern separation used non-meaningful stimuli, and found evidence of hippocampal involvement, but these either included items that resembled real objects and were verbally labeled (Duff et al., 2012), or required only perceptual (Mitchnick et al., 2022) or working memory discrimination (Paleja et al., 2014). Recently, Wammes et al. (2022) used fractals in a statistical learning paradigm and demonstrated that representational change of items with differing levels of similarity corresponds to a U-shaped function in the dentate gyrus. A U-shaped function would be predicted by the nonmonotonic plasticity hypothesis, so that the representation of only moderately similar items (but not low- or high-similarity items) was driven toward differentiation after learning relative to the pre-learning phase. This discrepancy between the results may be explained by the fact that in the study by Wammes et al. (2022), a relatively small set of fractals (16) were presented over a long sequence learning period, thus allowing participants to familiarize themselves well with each individual item. In fact, hippocampal repulsion seems to occur as a result of extensive learning (Chanales et al., 2017; Hulbert & Norman, 2015), and internal beliefs about the encoded stimuli may play a key role in triggering the repulsion of ambiguous inputs (Wanjia et al., 2025). In our study, each similar, interference inducing fractal was presented only once, and their hippocampal representation could not be stabilized through learning. It would be interesting to analyze the data of Wammes et al. (2022), from the beginning of the learning period, which would yield a closer correspondence between the two designs. To the best of our knowledge, ours is the first study to examine how interfering, non-meaningful stimuli are encoded in long-term, episodic-like memory through one-shot learning by the CA3DG (cf. Brickman et al., 2014).

Our results align with a representational account of the medial temporal lobe, that is, the view that areas are not specialized in terms of the type of computation they carry out, but rather according to the type of information they process (Cowell et al., 2019; Martin & Barense, 2023). Advocates of this account see a continuity between the visual and memory systems (Barense et al., 2005; Martin & Barense, 2023; Yonelinas, 2013), with the complexity of representable information gradually increasing from the early visual cortex to the hippocampus. Although the fractal objects were visually complex and the nature of the tasks required binding between locations and objects, the dimensionality of the stimuli was still below those of meaningful images, which can be ascribed multiple conceptual features as well as personal associations. For example, an image of a dog can be placed along dimensions of perceptual features, such as the color and luminosity of each pixel, and along semantic dimensions, such as the biological attributes of the species Canis familiaris, or emotions we associate with them. This expanded representational space of familiar objects has been shown to be also beneficial to working memory, relative to abstract visual items (Asp et al., 2021; Chung et al., 2024). Speculatively, it may be that the fractals did not elicit pattern separation in the hippocampus as images of everyday objects normally do because these stimuli were unfamiliar to participants and thus were comparably lower dimensional, lacking in contextual and meaningful features.

Early neuroimaging work already demonstrated that medial temporal lobe activity is modulated by meaningfulness (Martin et al., 1997), and later that the hippocampus responds with higher activation to familiar faces and real objects relative to novel ones during perceptual discrimination (Barense et al., 2011). There are several viable explanations for how hippocampal activity could be modulated by meaningfulness. First, semantic features contribute to object memorability, which in turn modulates hippocampal activation (Slayton et al., 2025). Second, as an analogue to spatial information processing, it has been suggested that the hippocampus constructs a conceptual cognitive map (Morton et al., 2017; Morton & Preston, 2021), represents semantic similarity between known places and people (Morton et al., 2021), and tracks distances in conceptual feature space, especially in dimensions relevant for actions and task performance (Barnaveli et al., 2025; Mack et al., 2016; Theves et al., 2020). Third, semantic information could be directly represented in the hippocampus, for example, by concept cells (De Falco et al., 2016; Mackay et al., 2024; Quiroga et al., 2008), although how concept cells relate to pattern separation is a contested question (Quiroga, 2020; Suthana et al., 2021).

To fully test the hypothesis that only experiences that differ in meaningful features are encoded in long-term memory by the CA3DG in a pattern separated fashion, the encoding of meaningful and non-meaningful stimuli in the hippocampus should be compared in a single mnemonic discrimination paradigm. Future studies could also shed light on whether making participants more familiar with non-meaningful stimuli (e.g., like Wammes et al., 2022), or assigning meaning and associations to them, may facilitate pattern separation for long-term memory in the hippocampus.

Outside of the MTL, we found evidence that the middle frontal gyrus and parietal regions are activated during the encoding of similar trials irrespective of information domain, and their connectivity increases within the network and with downstream sensory regions, when interference resolution is necessary. We interpret this as a regulation of regions that process sensory information in order to either emphasize or downplay differences. These data alone cannot disambiguate, however, whether the connectivity increase facilitated the integration or differentiation of interfering information in this context. A subsequent memory analysis could shed light on how the activation of these regions contributed to memory performance. Our design did not allow for this type of analysis, because each recognition trial can be paired with two encoding trials. However, our behavioral results do show that both the similarity of the first and second presentation encoding trials affected participants recognition performance, indicating that participants did not only “encounter” similar trials, but also encoded them. Thus, the activation of frontal and parietal regions during similar trials suggests their involvement in encoding interfering stimuli.

The whole-brain gPPI analysis did not provide evidence that frontal and parietal connectivity with the MTL changed during the encoding of similar trials. This could be related to either the relatively low power of PPI methods for fMRI data (Di & Biswal, 2017; O’Reilly et al., 2012), the lower signal-to-noise ratio and small volume of MTL regions, or a stable connectivity profile between the MTL and the frontoparietal areas. It is important that the direction and amount of influence of the frontoparietal regions may not be directly translatable into changes in functional connectivity, and influence could also be exerted over stable connectivity, which led us to examine the region-to-region connectivity specifically between the MTL and frontoparietal ROIs.

Our region-to-region connectivity analysis between MTL and frontal and parietal seeds confirmed functional connectivity between these networks. We found some evidence that the connectivity between the PHC changed according to task context (Object or Location), however, this result was not significant after correction for multiple comparisons, and no other differences were found in the connectivity patterns of the two tasks. Altogether, these results indicate an involvement of frontoparietal regions in resolving interference, nonetheless, more evidence is needed to elucidate whether and how these regions interact with the MTL to achieve interference resolution. It also remains an open question how activity in frontoparietal regions impacts interference resolution in the MTL

We chose to analyze the neural signals of pattern separation at the time of encoding in order to reduce the contaminating effects of retrieval, in accordance with previous theoretical suggestions. There is also some empirical evidence indicating that lure discrimination at retrieval may be confounded by pattern completion and a recall to reject strategy (Molitor et al., 2014; Wahlheim & Richmond, 2025). However, neither encoding nor retrieval tasks using similar lures are process pure (Ngo et al., 2021), and we cannot completely rule out the possibility that pattern completion and recognition processes took place during encoding, despite the use of fractals. It is in fact likely that participants recognized features of repeat and similar fractals already during study, which may be also reflected in the activation of retrieval-related areas, such as the angular gyrus, in the whole-brain results. However, participants were instructed to pay attention to the details of each trial (specifically fractal identity in the Object Task and fractal location in the Location Task) in order to commit them to their memory, and thus were encouraged to engage in the encoding, rather than retrieval of trials.

A limitation of this study is the combined analysis of the CA3 and dentate gyrus areas. Given the role of CA3 in pattern completion, one might argue that the activity pattern we see in the CA3DG is the result of pattern completion in the CA3. At the time of analyzing these data, there was no published consensus-based, valid and reliable method of delineating these two subfields on 3T MRI data (Olsen et al., 2019). In collapsing the two subfields, we have followed a substantial body of previous fMRI studies that nonetheless found a pattern separation signal in this combined region, albeit using everyday objects as stimuli (Chanales et al., 2017; Dimsdale-Zucker et al., 2018; Favila et al., 2016; Reagh & Yassa, 2014; Yassa & Stark, 2011). Note, however, that a harmonized segmentation protocol of the hippocampal body that defines the boundary between the CA3 and DG has been recently developed and is now available as a preprint (Daugherty et al., 2025). Based on this exciting new development, we hope that future studies will elucidate the dynamics of pattern separation in both the CA3 and DG—potentially through the reanalysis of this dataset as well.

Our conclusions regarding the extent of domain-selective functional specialization in the MTL are limited by the lack of entorhinal cortex (EC) results. Numerous previous studies suggested that the anterolateral entorhinal cortex is engaged in object processing, and the posterior medial entorhinal cortex is engaged in spatial processing (Berron et al., 2018; Knierim et al., 2014; Reagh & Yassa, 2014), and this view is supported by functional connectivity results (Maass et al., 2015). Unfortunately, the low signal-to-noise ratio in this area in our fMRI dataset made it infeasible to draw any conclusions about domain-specific processing in the EC. Future neuroimaging work, with acquisition parameters better suited to study this area, should shed light on the domain selectivity of the anterolateral and posterior medial parts of the EC, and how these interact with other MTL regions.

To conclude, our work critically revisits the question of pattern separation in the CA3DG and domain-dependent interference reduction in the medial temporal neocortex. We replicated findings on content-specific processing in the MTL with non-meaningful stimuli and provided evidence that frontoparietal regions contribute to mnemonic discrimination. Contrary to our expectations, we found no evidence that the CA3DG discriminates between repeats and interference-inducing similar stimuli. These results advance our understanding of how unique memories are created by a widespread collaboration of brain areas and raise the intriguing question where the limits of hippocampal pattern separation lie.

## Supplementary Material

Supplementary Material

## Data Availability

Neuroimaging data: https://openneuro.org/datasets/ds005559/ Behavioral data: https://osf.io/q9wks/?view_only=93849d7075374aad8dfce5b6c69e4e8a Analysis code: https://github.com/zs-nemecz/miniTRK

## Supplementary Materials

Supplementary material for this article is available here: https://osf.io/uzgx6
