# Supplementary material for: fMRI signals of pattern separation in the neocortex and hippocampus to non-meaningful objects and their spatial location
Source: Imaging Neurosci (Camb). 2026 Jan 16;4:IMAG.a.1088. doi: 10.1162/IMAG.a.1088 (PMC12813878; doi:10.1162/IMAG.a.1088)
Supplement: Supplementary Material [file IMAG.a.1088_supp.pdf]

Supplementary Information

fMRI signals of pattern separation in the neocortex and hippocampus to non-meaningful objects and their spatial location

Zsuzsanna Nemecz[1,2,3], István Homolya[3,4], Alex Ilyés[1,2,3], Hunor Kis[1,2,3], György Mező[5,6], Virág Anna Varga[2,3], Markus Werkle-Bergner[7], Attila Keresztes[3,2]

1 Doctoral School of Psychology, ELTE Eötvös Loránd University, 1064 Budapest, Hungary
2 Institute of Psychology, ELTE Eötvös Loránd University, 1064 Budapest, Hungary
3 Brain Imaging Centre, HUN-REN Research Centre for Natural Sciences, 1117 Budapest, Hungary
4 Budapest University of Technology and Economics, 1111 Budapest, Hungary
5 Wigner Datacenter, HUN-REN Wigner Research Centre for Physics, 1121 Budapest, Hungary
6 Konkoly Observatory, HUN-REN Research Centre for Astronomy and Earth Sciences, 1121 Budapest, Hungary
7 Center for Lifespan Psychology, Max Planck Institute for Human Development, 14195 Berlin, Germany

Correspondence: Zsuzsanna Nemecz (nemecz.zsuzsanna@ppk.elte.hu)

Table of contents

Supplementary Materials....0

Supplementary Results....2

Models of behavioral sensitivity to the first and second object similarity....2

Supplementary Table 1. The effect of similarity on the ratio of “new” responses in the Object Task....2

Supplementary Table 2. Model Comparisons - The effect of object feature similarity on the ratio of new responses in the Object Task....2

Models of behavioral sensitivity to the first and second spatial similarity....2

Supplementary Table 3. The effect of similarity on the ratio of “new” responses in the Location Task....2

Supplementary Table 4. Model Comparisons - The effect of spatial similarity on the ratio of new responses in the Location Task....4

Associations between behavior and neuronal sensitivity to similarity....4

Supplementary Figure 1. The association between the right CA3DG sensitivity to spatial similarity and behavioral sensitivity to spatial sensitivity....4

Modeling Baseline (multiple repetition) trials....5

Supplementary Figure 2....5

Whole-brain analysis: (Similar - First) > (Repeats - First) contrast results....6

Supplementary Table 4. Significant clusters for contrast (Similar - First) > (Repeats - First) during Object Encoding....6

Supplementary Table 5. Significant clusters for contrast (Similar - First) > (Repeats - First) during Location Encoding....6

Whole-brain analysis: Similar - First contrast results....7

Supplementary Table 6. Significant clusters for the contrast Similar - First during Object Encoding....7

Supplementary Table 7. Significant clusters for the contrast Similar - First during Location Encoding....7

Neurosynth Analysis....8

Supplementary Table 8. Object Similar > First. Top 10 Results....8

Supplementary Table 9. Object Similar > First. Memory related terms....8

Supplementary Table 10. Location Similar > First. Top 10 Results....8

Supplementary Table 11. Location Similar > First. Memory related terms....9

Connectivity between the frontoparietal and medial temporal regions....9

Supplementary Figure 3....9

Supplementary Figure 4....10

Associations between behavior and frontoparietal-medial temporal connectivity....11

Supplementary Table 12. Object mnemonic discrimination and frontoparietal-medial temporal connectivity....11

Supplementary Table 13. Location mnemonic discrimination and frontoparietal-medial temporal connectivity....11

Supplementary Methods....12

Stimuli assignment to conditions....12

Supplementary Figure 5. Structure of the Object task....12

Supplementary Figure 6. Structure of the Location task....12

Spatial Similarity Manipulation....13

Supplementary Figure 7 13
Object Similarity Rating Experiment 13
Supplementary Figure 8 14
Segmentation of region of interests in the medial temporal lobe 14
Supplementary Figure 9. Medial temporal ROIs on an example segmentations. 14
Registration of partial FOV high-resolution PD images to functional scans 15
Supplementary Figure 10. Example ROI to Functional registrations 16
Mixed effects model assumptions 17
Behavioral Models: Generalized binomial mixed effects models 17
Neural Models: Linear mixed effects models 17
Supplementary Figure 11 17
References 18

Supplementary Results

Models of behavioral sensitivity to the first and second object similarity

Supplementary Table 1. The effect of similarity on the ratio of "new" responses in the Object Task.

Model	Fixed effects	Estimate	Std Error	*z*	*p*	AIC
**A**	*FirstSimilarity*	0.16	0.02	9.23	< .001	2562.93
**B**	*SecondSimilarity*	0.17	0.02	8.98	< .001	2565.45
**C**	*FirstSimilarity*	0.02	0.06	0.3	.76	2545.91
	SecondSimilarity	0.09	0.03	3.41	< .001	
	*FirstS. * SecondS.*	0.02	0.01	1.64	.1	

Supplementary Table 2. Model Comparisons - The effect of object feature similarity on the ratio of new responses in the Object Task

Models	N par.	AIC	BIC	Log Likelihood	Deviance	Chi-square	df	*p*
**A**	3	2562.9	2579.8	-1278.5	2556.9		2	< .001
**C**	5	2545.9	2574.1	-1268.0	2535.9	21.02		
**B**	3	2565.5	2582.4	-1279.7	2559.5			< .001
**C**	5	2545.9	2574.1	-1268.0	2535.9	23.55	2	

Models of behavioral sensitivity to the first and second spatial similarity

Supplementary Table 3. The effect of similarity on the ratio of "new" responses in the Location Task.

Model	Fixed effects	Estimate	Std Error	*z*	*p*	AIC
**A**	*FirstSimilarity*	0.11	0.02	7	< .001	4242.24
**B**	*SecondSimilarity*	0.12	0.02	6.5	< .001	4249.73
**C**	*FirstSimilarity*	0.23	0.05	4.53	< .001	4233.07
	SecondSimilarity	0.11	0.04	2.74	.006	
	*FirstS. * SecondS.*	-0.03	0.01	-3.49	< .001	

Supplementary Table 4. Model Comparisons - The effect of spatial similarity on the ratio of new responses in the Location Task

Models	N par.	AIC	BIC	Log Likelihood	Deviance	Chi-square	df	*p*
**A**	3	4242.2	4260.4	-2118.1	4236.2			
**C**	5	4233.1	4263.3	-2111.5	4223.1	13.16	2	.001
**B**	3	4249.7	4267.8	-2121.9	4243.7			
**C**	5	4233.1	4263.3	-2111.5	4223.2	20.67	2	< .001

Associations between behavior and neuronal sensitivity to similarity

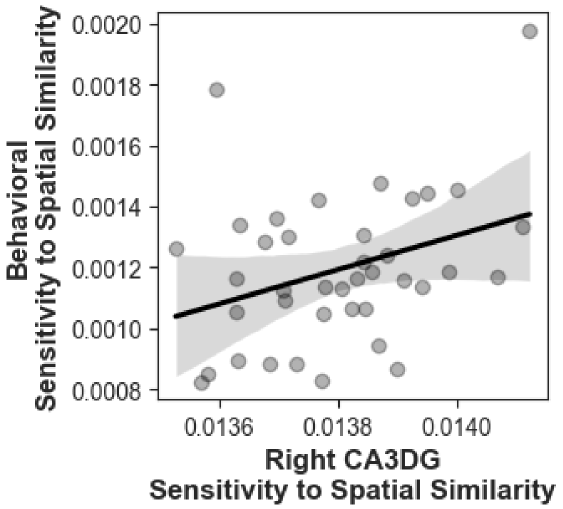

Supplementary Figure 1. The association between the right CA3DG sensitivity to spatial similarity and behavioral sensitivity to spatial sensitivity. The association was not significant after correcting for multiple comparisons ($r = 0.34$, $p_{unadjusted} = .034$).

Modeling Baseline (multiple repetition) trials

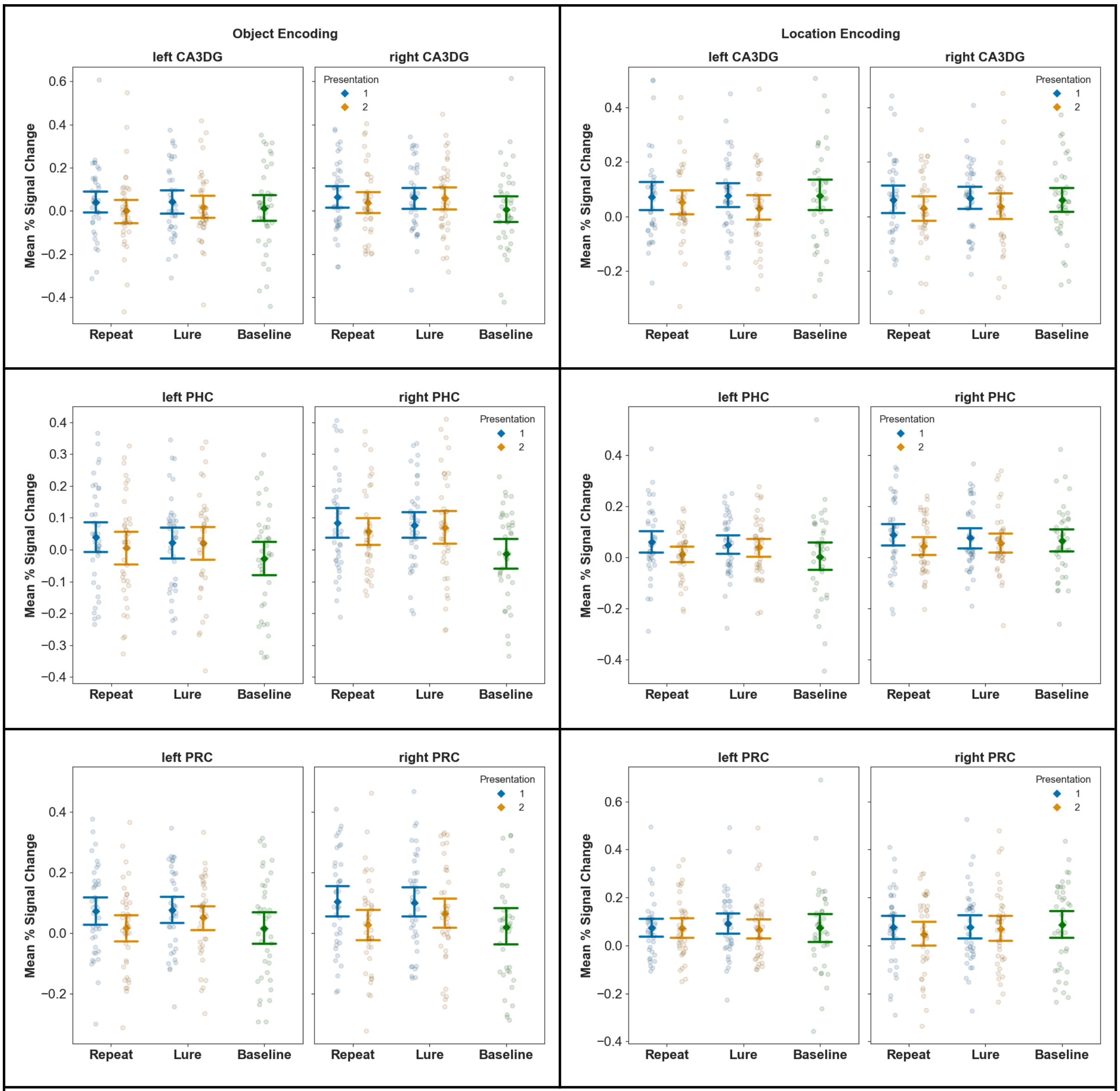

Supplementary Figure 2.

Whole-brain analysis: (*Similar - First*) > (*Repeats - First*) contrast results

Supplementary Table 4. Significant clusters for contrast (*Similar - First*) > (*Repeats - First*) during Object Encoding

Area	N voxels	Max. Z value	*p*
bilateral lateral occipital cortex, occipital fusiform gyrus, right inferior temporal gyrus	25676	4.97	< .001
right inferior frontal gyrus, right precentral gyrus, right middle frontal gyrus	1609	3.79	< .001
left lateral occipital cortex	1456	3.52	< .001
right lateral occipital cortex	1349	3.63	< .001
right postcentral gyrus and right supramarginal gyrus	980	3.27	< .001
left frontal pole, left inferior frontal gyrus (pars triangularis)	891	3.45	< .001
bilateral paracingulate cortex	821	3.51	< .001
left inferior frontal gyrus (pars opercularis)	723	3.85	< .001
right postcentral gyrus, and right superior parietal lobule	596	3.26	< .001
right frontal operculum and insular cortex, right putamen	595	3.23	< .001
left postcentral gyrus and left supramarginal gyrus	542	3.33	< .001
left precentral gyrus and middle frontal gyrus (frontal eye field)	488	3.49	< .001
right frontal pole	473	3.87	< .001
left postcentral gyrus and left supramarginal gyrus	449	3.56	< .001
left superior parietal lobule	405	3.37	.002
bilateral supplementary motor area	289	3.4	.034

Supplementary Table 5. Significant clusters for contrast (*Similar - First*) > (*Repeats - First*) during Location Encoding

Area	N voxels	Max. Z value	*p*
bilateral lingual gyrus, bilateral occipital fusiform gyrus, bilateral occipital pole and lateral occipital cortex	8124	4.46	< .001

Whole-brain analysis: *Similar - First* contrast results

Supplementary Table 6. Significant clusters for the contrast *Similar - First* during Object Encoding

Area	N voxels	Max. Z value	*p*
right frontal pole, right middle frontal gyrus, right orbitofrontal cortex, bilateral paracingulate gyrus	31370	5.91	< .001
bilateral precuneus	15531	5.63	< .001
right lateral occipital cortex, right angular gyrus, right supramarginal gyrus	13958	6.2	< .001
left lateral occipital cortex, left supramarginal gyrus, left angular gyrus	8268	5.18	< .001
left middle frontal gyrus	6515	4.87	< .001
left frontal pole	4505	5.18	< .001
right middle temporal gyrus (posterior division)	4051	4.38	< .001
left middle temporal gyrus, posterior division	1552	4.26	< .001
bilateral occipital pole and lingual gyrus	1466	3.46	< .001
bilateral posterior cingulate	970	4.34	< .001
left cerebellum	665	5.41	< .001
left orbitofrontal cortex	647	4.27	< .001
right cerebellum	301	3.55	.034

Supplementary Table 7. Significant clusters for the contrast *Similar - First* during Location Encoding

Area	N voxels	Max. Z value	*p*
right angular gyrus, right supramarginal gyrus, bilateral precuneus, lingual gyrus, occipital pole, bilateral lateral occipital cortex	44544	5.68	< .001
right middle frontal gyrus	9691	4.71	< .001
left angular gyrus, supramarginal gyrus, left lateral occipital cortex	5509	4.26	< .001
left middle frontal gyrus	5457	4.28	< .001
right frontal pole	5156	4.6	< .001
left frontal pole	3657	4.44	< .001
right superior and middle temporal gyrus	2316	4.19	< .001
bilateral superior frontal gyrus and paracingulate gyrus	1924	04.04	< .001
left superior, middle, and inferior temporal gyrus	944	3.67	< .001
left cerebellum	553	4	< .001
right frontal pole	456	3.48	< .001
right orbitofrontal cortex	360	3.57	.007

Neurosynth Analysis

Supplementary Table 8. Object Similar > First. Top 10 Results.	
**Neurosynth term**	**Correlation**
parietal	0.199
inferior parietal	0.189
dorsolateral	0.183
working memory	0.166
working	0.164
parietal	0.164
precuneus	0.158
dorsolateral prefrontal	0.146
frontoparietal	0.143
task	0.138

Supplementary Table 9. Object Similar > First. Memory related terms.	
**Neurosynth term**	**Correlation**
default mode	0.026
memory retrieval	0.104
recognition	0.07
episodic memory	-0.001

Supplementary Table 10. Location Similar > First. Top 10 Results.	
**Neurosynth term**	**Correlation**
parietal	0.232
precuneus	0.189
parietal cortex	0.188
working memory	0.183
working	0.181

inferior parietal	0.167
cuneus	0.158
frontoparietal	0.154
dorsolateral	0.138
memory	0.136

**Supplementary Table 11. Location Similar > First. Memory related terms.**	
**Neurosynth term**	**Correlation**
default mode	0.042
memory retrieval	0.09
recognition	0.06
episodic memory	0.014

Connectivity between the frontoparietal and medial temporal regions

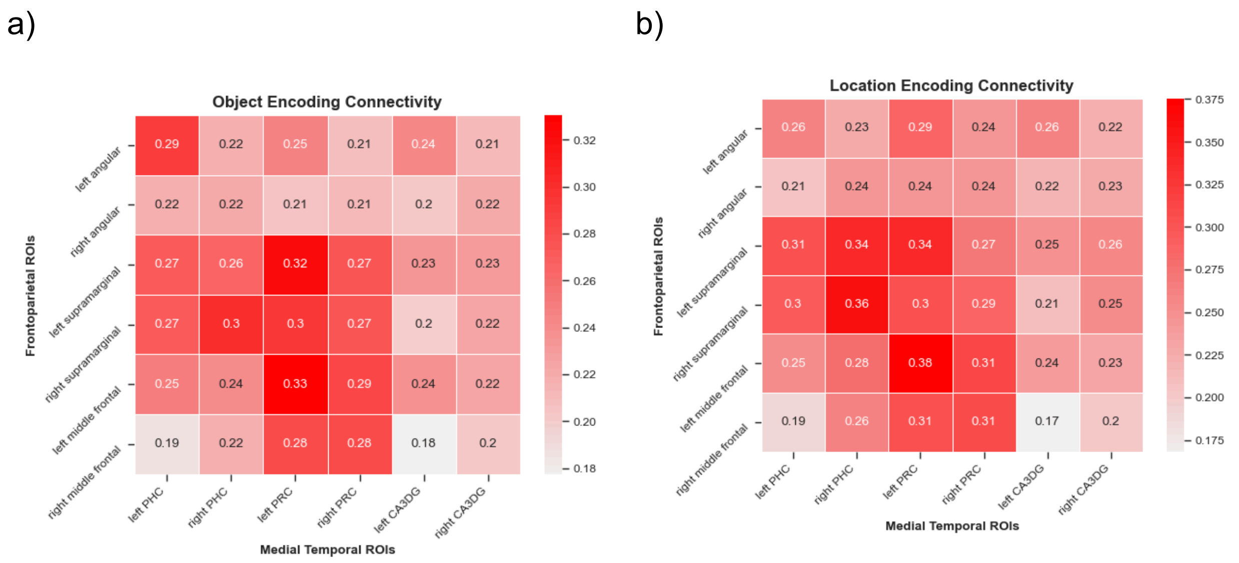

Supplementary Figure 3.

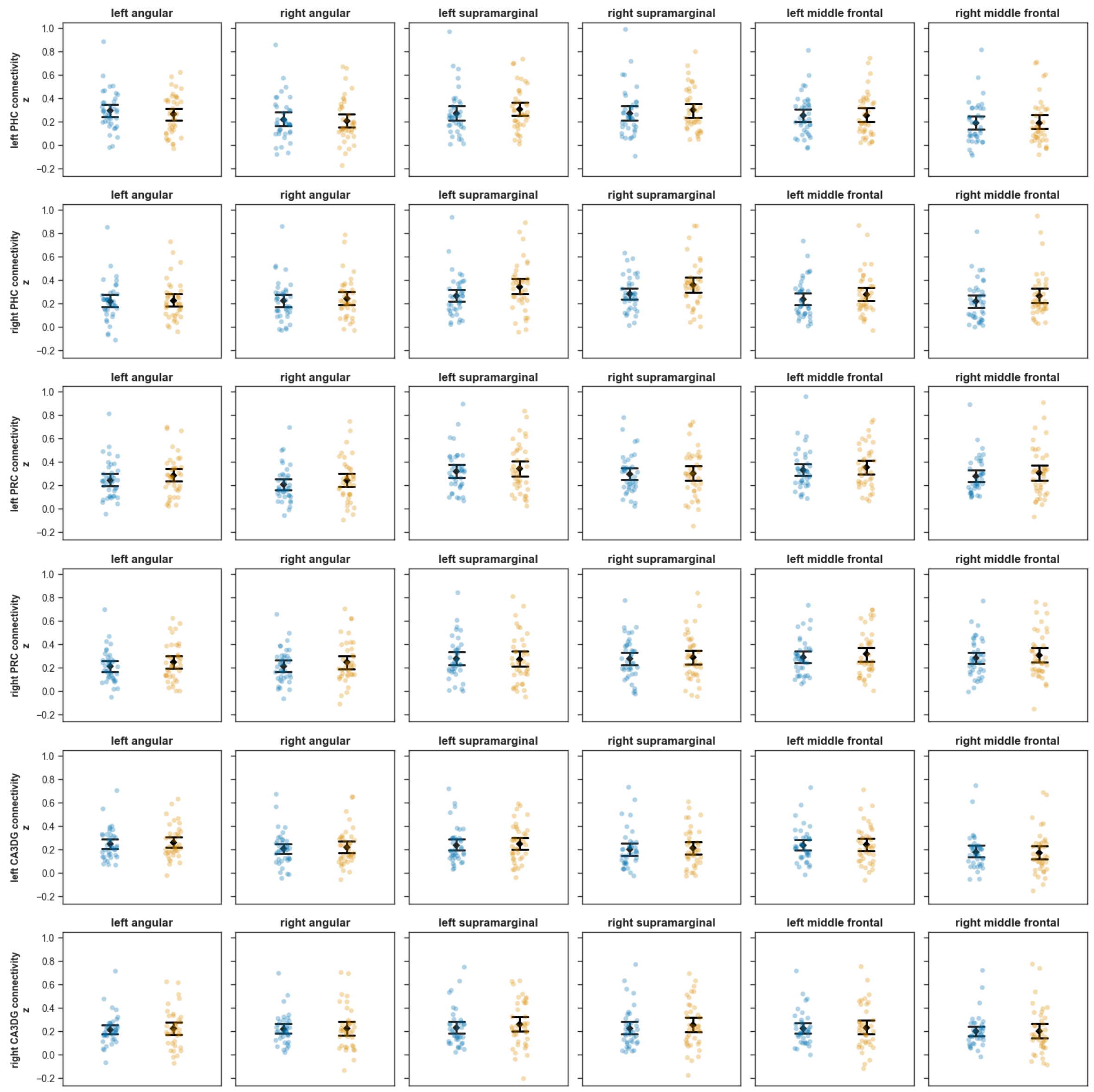

Supplementary Figure 4.

Associations between behavior and ROI-to-ROI connectivity

Of interest, in the Object task the sensitivity to the similarity between the first encoding presentation and the recognition trial was associated with the connectivity between the right PRC and right angular gyrus (r = -0.41, p = .01). The sensitivity to the similarity between the second encoding presentation and the recognition trial was associated with the connectivity between the right PHC and left supramarginal gyrus (r = -0.41, p = .009), and the left PRC and right supramarginal gyrus (r = -0.41, p = .009). In the Location task the associations were weaker, but also exclusively negative, and included both the PRC and PHC connectivity (see Supplementary Table 13).

Supplementary Table 12. Object mnemonic discrimination and frontoparietal-medial temporal connectivity

Similarity Sensitivity	Task	MTL ROI	Frontoparietal ROI	Spearman r	p
SecondSimilarity	Object	left PRC	right supramarginal	-0.41	0.001
SecondSimilarity	Object	right PHC	left supramarginal	-0.41	0.001
FirstSimilarity	Object	left PRC	right angular	-0.41	0.001
FirstSimilarity	Object	right PHC	left angular	-0.39	0.012
SecondSimilarity	Object	right PHC	right supramarginal	-0.39	0.014
SecondSimilarity	Object	left PHC	left supramarginal	-0.38	0.018
FirstSimilarity	Object	right PHC	right angular	-0.37	0.021
SecondSimilarity	Object	left PRC	left supramarginal	-0.36	0.023
FirstSimilarity	Object	right PHC	left middle frontal	-0.34	0.032
FirstSimilarity	Object	left PHC	right angular	-0.34	0.033
SecondSimilarity	Object	left PHC	right supramarginal	-0.33	0.037
FirstSimilarity	Object	right PRC	right middle frontal	-0.33	0.038
SecondSimilarity	Object	left PHC	left middle frontal	-0.33	0.04
FirstSimilarity	Object	left PRC	left angular	-0.32	0.044
FirstSimilarity	Object	left PHC	left angular	-0.32	0.049

Supplementary Table 13. Location mnemonic discrimination and frontoparietal-medial temporal connectivity

Similarity Measure	Task	MTL ROI	Frontoparietal ROI	Spearman r	p
FirstSimilarity	Location	left PRC	right middle frontal	-0.38	0.017
FirstSimilarity	Location	right PHC	right angular	-0.35	0.03
FirstSimilarity	Location	right PHC	right middle frontal	-0.32	0.045

Interpretation of the associations between behavior and connectivity

The associations between the MTL-frontoparietal connectivity and recognition performance suggest that the synchronicity of these areas carries behavioral relevance. Previously, suppression of competing memory patterns in ventral visual areas by the lateral prefrontal cortex has been shown to lead to adaptive forgetting of interfering information (Wimber et al., 2015), and numerous studies demonstrated the role of the dorsolateral prefrontal cortex in reducing interference by retrieval suppression and inhibitory control (Anderson et al., 2004; Anderson & Hulbert, 2021; Depue et al., 2016). Based on these findings, it may be that the MTL - frontoparietal connectivity contributed to the suppression of competing information, which in turn led to the decreased sensitivity to similarity.

Supplementary Methods

Stimuli assignment to conditions

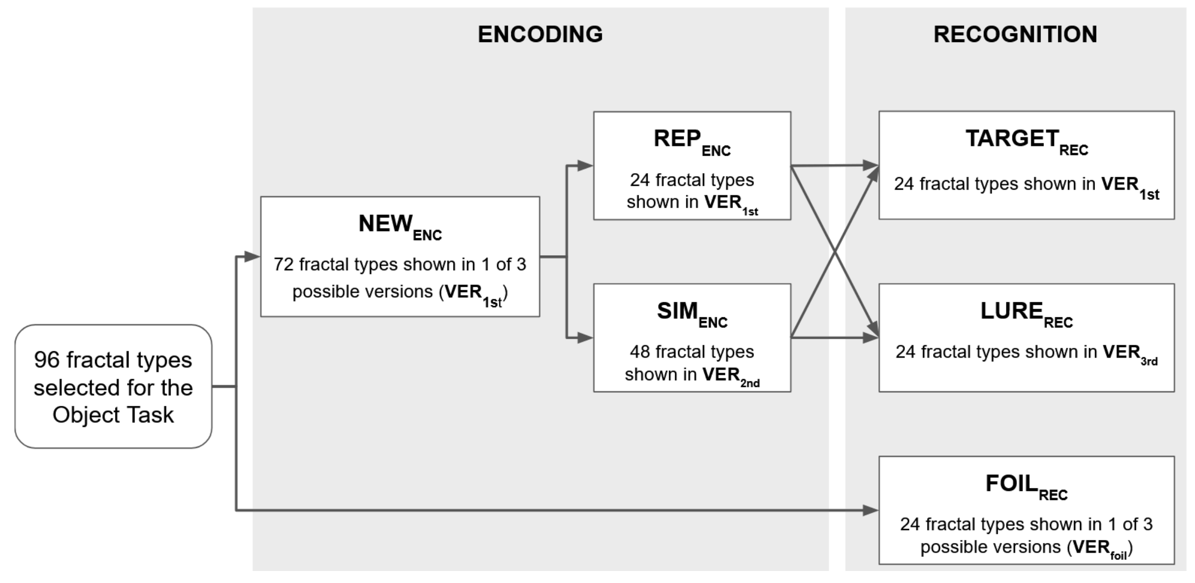

Supplementary Figure 5. Structure of the Object task

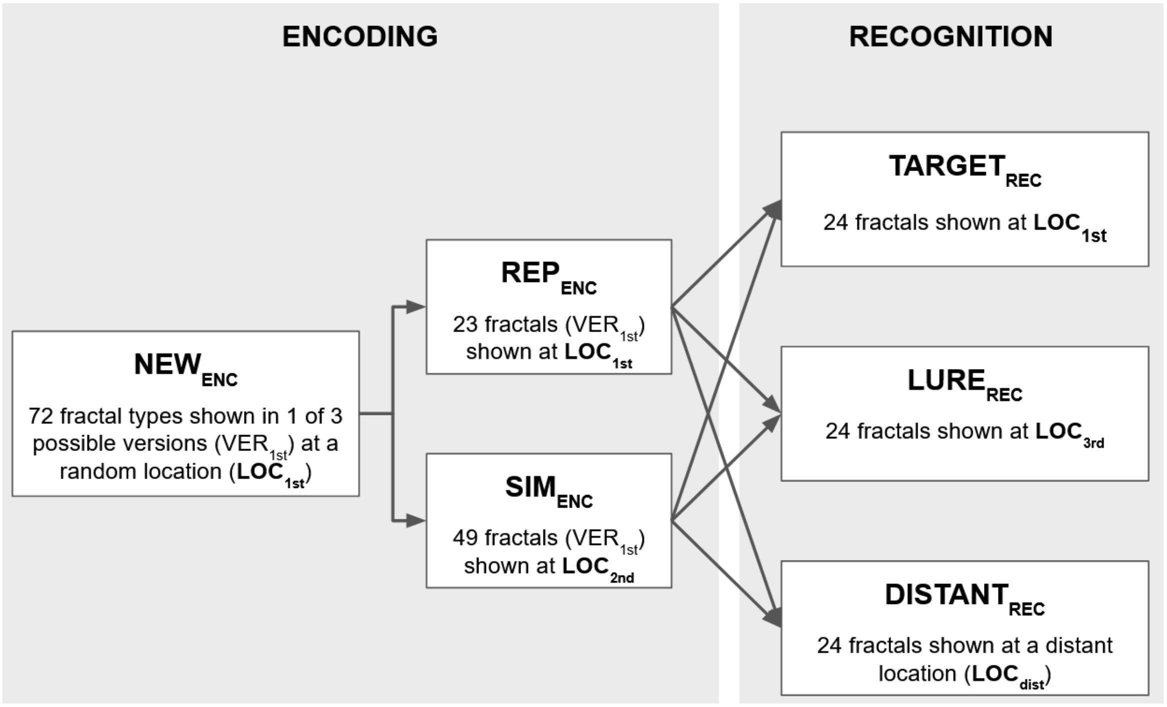

Supplementary Figure 6. Structure of the Location task

Spatial Similarity Manipulation

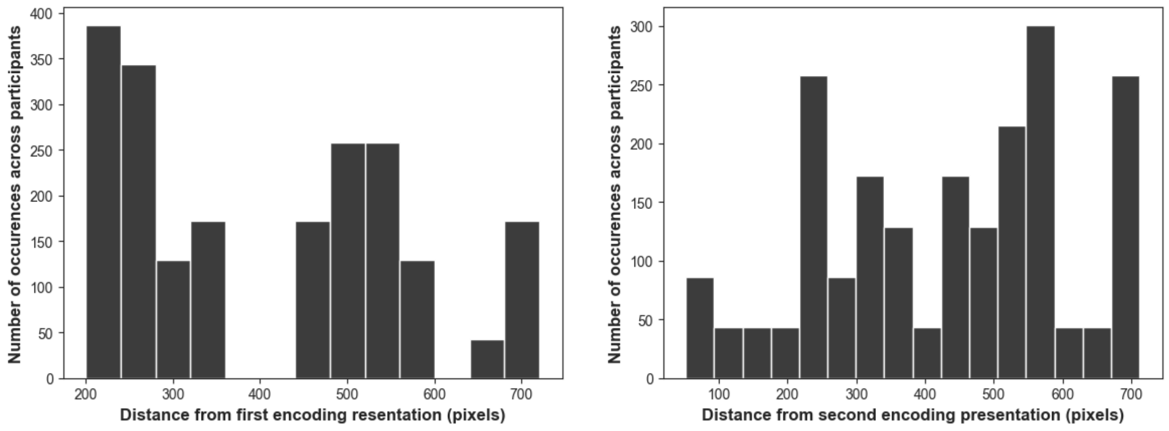

Supplementary Figure 7.

Object Similarity Rating Experiment

Thirty participants were recruited from the local student community to rate the similarity between the three versions of fractal types. Participants were screened for color blindness with a short Ishihara Test (Ishihara, 1951) consisting of 5 plates. The experiment was programmed with Psytoolkit 3.4.0 (Stoet, 2010, 2017). Participants were

randomly assigned to 3 groups, and each group rated a non-overlapping set of fractal pairs, so that overall the three groups rated all pairs of fractal versions within each fractal type (e.g., Group A rated the pair 1a - 1b, Group B rated 1b - 1c, and Group C rated 1a - 1c). In each trial, participants were presented with a pair of images, showing 2 different versions of the same fractal type. Participants had a maximum of 10 seconds to rate the similarity of the two images on a scale from 0 (identical) to 9 (very different). Rating trials were preceded by 20 practice trials, after which participants received feedback on their average rating, and were encouraged to use the whole extent of the scale. Participants completed 200 rating trials. In 19 of the rating trials, 2 identical fractal images were shown, which were not used as stimuli in the mnemonic discrimination task. The experimental session lasted 15-20 minutes, and participants were compensated with course credits.

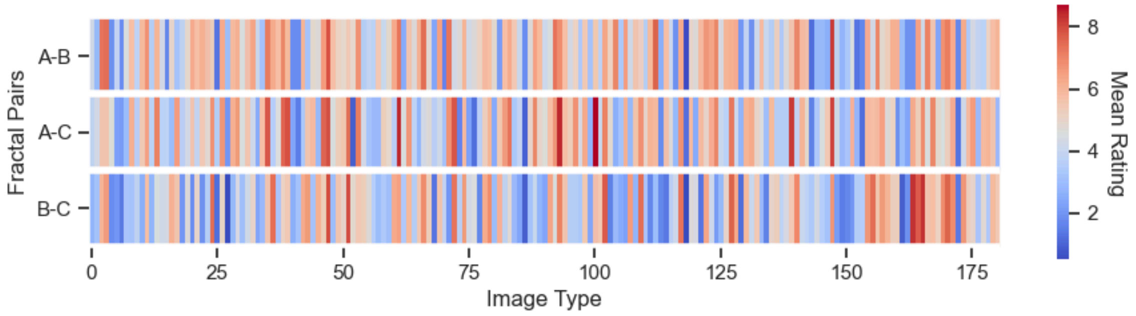

Supplementary Figure 8. Mean similarity rating between the fractal versions (A-B-C) of each Image Type (0-180).

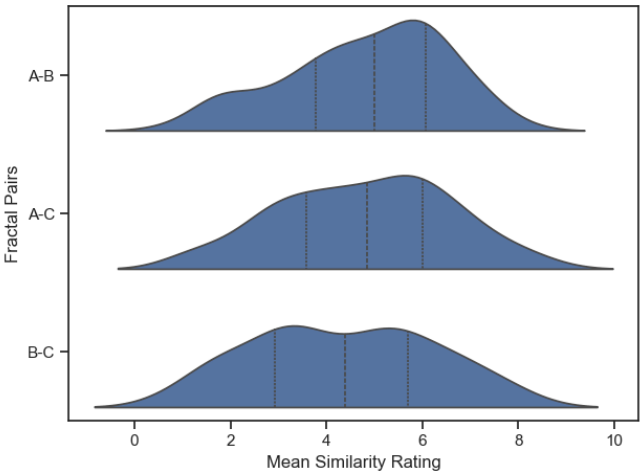

Supplementary Figure 9. Violin plots of the mean similarity ratings between fractal pairs. The dashed line in the middle indicates the median. Dotted lines on the left and right indicate the lower and upper quartiles.

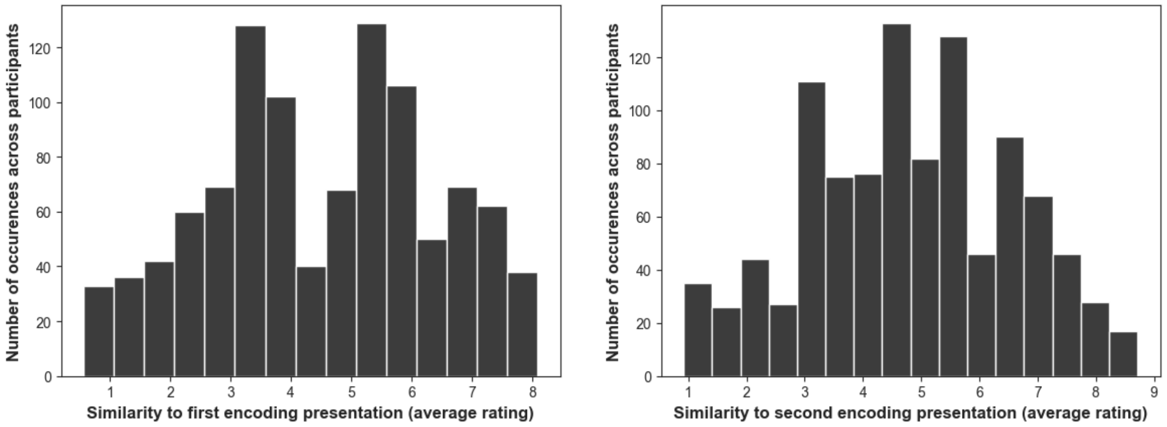

Supplementary Figure 10.

Segmentation of region of interests in the medial temporal lobe

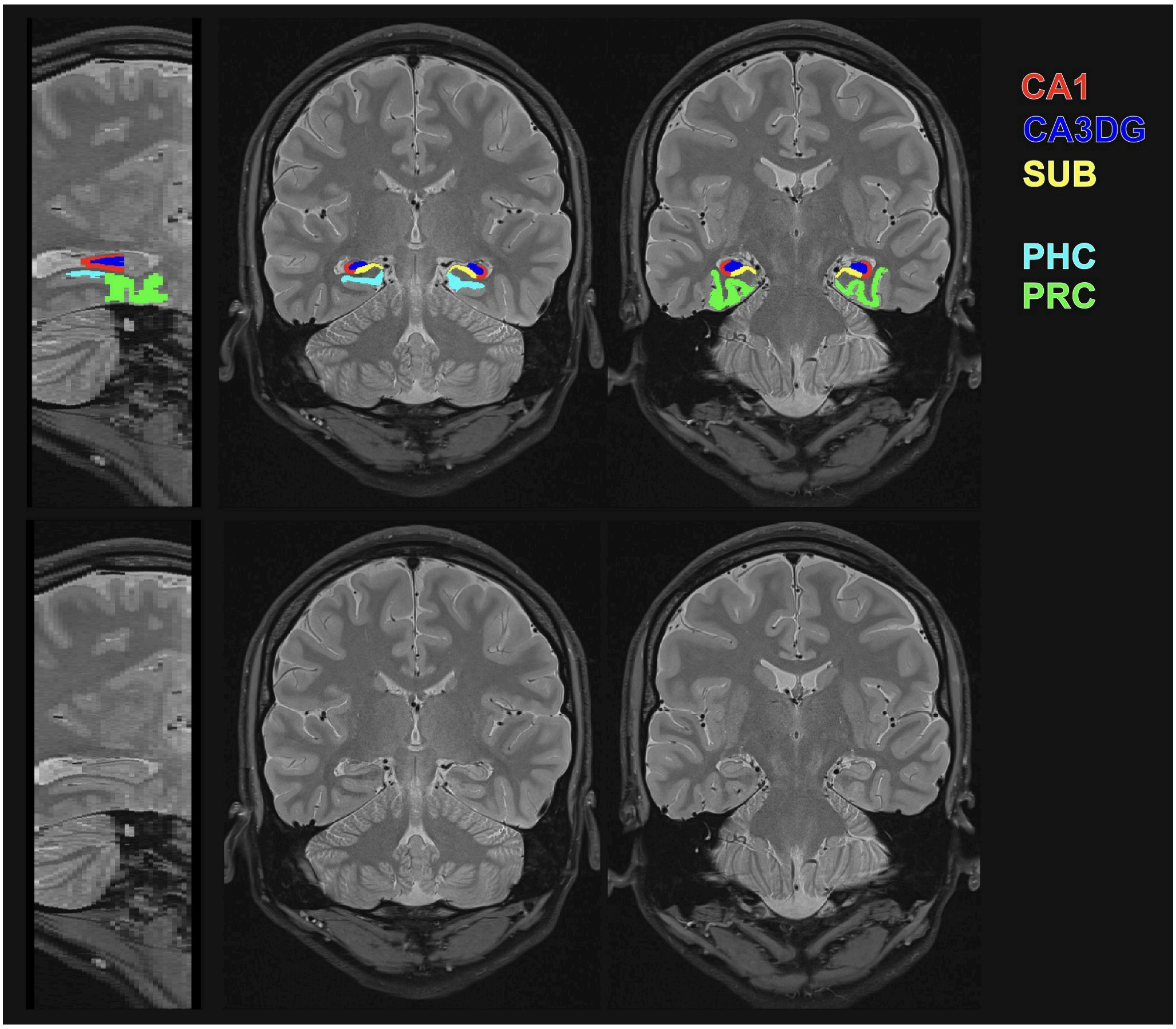

Supplementary Figure 11. Medial temporal ROIs on an example segmentation. The depicted segmentations of hippocampal subfields (CA1, CA3DG, SUB) are already manually corrected following Canada et al. (2023).

Correction of automatic segmentations

Supplementary Table 14. The number of participants who needed any correction and the types of errors that occurred in the automatic segmentations of hippocampal subfields.

	left			*right*		
	CA1-2	CA3DG	SUB	CA1-2	CA3DG	SUB
Any error	4	6	5	6	6	4
Lack of label	0	0	0	0	0	0
Underestimated label	0	1	1	0	2	0
Overestimated label	0	0	0	1	0	1
Dropped pixels	4	4	4	5	4	4
Sulcal cavity	0	1	0	0	0	0

Registration of partial FOV high-resolution PD images to functional scans

We used the following three ANTs calls.

Step 1. Individual T1 weighted images were registered to the partial FOV high resolution PD images with the ANTs call:

```
antsRegistrationSyN.sh \
  -d 3 \
  -f highres_PD.nii.gz \
  -m T1.nii.gz \
  -t a \
  -o T1-To-hPD_
```

Step 2. The mean of the first Object Encoding functional run was registered to the individual T1 weighted anatomy with the ANTs command:

```
antsRegistration \
  --dimensionality 3 \
  --float 0 \
  --output [meanFunc-To-T1_, meanFunc-To-T1_Warped.nii.gz] \
  --interpolation Linear \
  --winsorize-image-intensities [0.005,0.995] \
  --use-histogram-matching 0 \
  --initial-moving-transform [T1.nii.gz, meanFunc.nii.gz, 1] \
  --transform Rigid[0.1] \
```

```
  --metric MI[T1.nii.gz, meanFunc.nii.gz,1,32,Regular,0.20] \
  --convergence [1000x500x100x0,1e-6,10] \
  --shrink-factors 8x4x2x1 \
  --smoothing-sigmas 3x2x1x0vox \
  --transform SyN[0.1,3,0] \
  --restrict-deformation 1x1x0 \
  --metric CC[T1.nii.gz, meanFunc.nii.gz,1,4] \
  --convergence [10,1e-6,10] \
  --shrink-factors 1 \
  --smoothing-sigmas 0vox  \
  --x MTL_mask.nii.gz \
```

Step 3. Medial temporal ROI masks were transformed to the functional space using the transformation matrices and warp image resulting from the above registrations:

```
antsApplyTransforms \
  -d 3 \
  -i ROI_mask.nii.gz\
  -t [meanFunc-To-T1_0GenericAffine.mat, 1] \
  -t meanFunc-To-T1_1InverseWarp.nii.gz \
  -t [T1-To-hPD_0GenericAffine.mat, 1] \
  -n GenericLabel \
  -o ROI-To-meanFunc.nii.gz
```

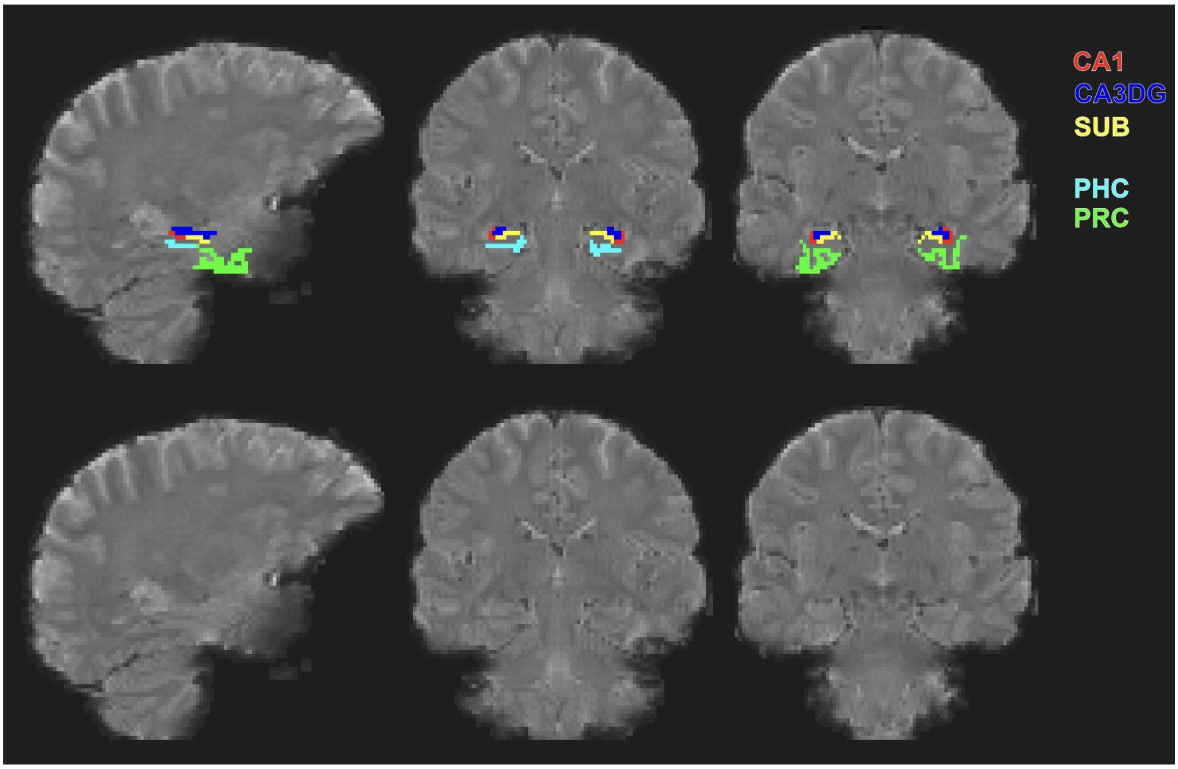

Supplementary Figure 12. Example ROI to Functional registrations

Mixed effects model assumptions

Model assumptions were tested using the performance 0.12.3 (Lüdecke et al., 2021) and EnvStat 3.0.0 (Millard, 2013) packages in R.

Behavioral Models: Generalized binomial mixed effects models

In the behavioral analysis, all modeled responses were binary (new response: 0/1). No outliers were found that have biased the models. Binned residual checks indicated that over 80% of residuals of all models were inside the error bounds, except for Model B of the Object Task (78% of residuals were inside the error bounds). We found no significant overdispersion in any of the models.

Neural Models: Linear mixed effects models

Q-Q Plots and residual plots indicated that residuals were normally distributed with homogenous variance across groups (Suppl. Fig 9).

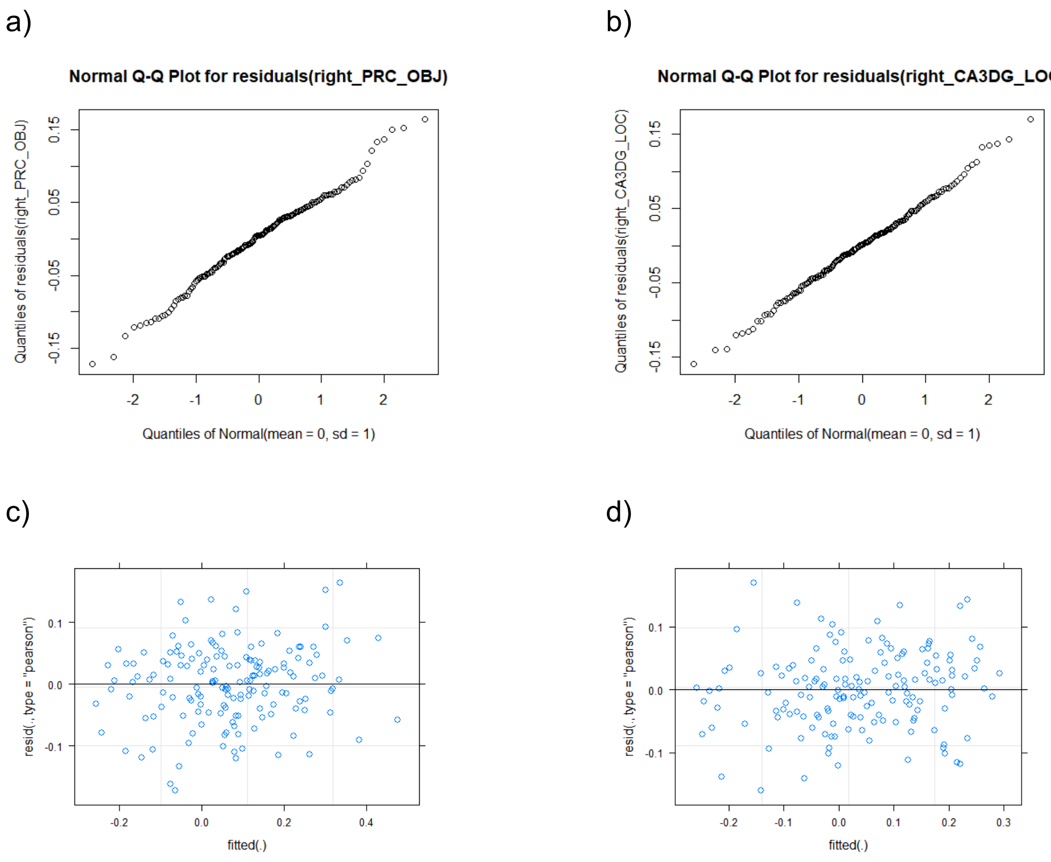

Supplementary Figure 13.
Q-Q Plots indicated that the residuals from models relating neural responses to similarity were normally distributed. a) Q-Q plot of the model predicting the response of the right PRC based on object similarity. b) Q-Q plot of the model predicting the response of the right CA3DG based on spatial similarity. Plotting standardized residuals against the predicted values suggests that the assumption of homoscedasticity was met in both models. c) Residuals from the right PRC response ~ object similarity model. d) Residuals from the right CA3DG response ~ spatial similarity model.

References

Canada, K. L., Mazloum-Farzaghi, N., Rådman, G., Adams, J. N., Bakker, A., Baumeister, H., Berron, D., Bocchetta, M., Carr, V., Dalton, M. A., Flores, R. de, Keresztes, A., Joie, R. L., Mueller, S. G., Raz, N., Santini, T., Shaw, T., Stark, C. E. L., Tran, T. T., … Group, the H. S. (2023). *A (Sub)field Guide to Quality Control in Hippocampal Subfield Segmentation on High-resolution T2-weighted MRI* (p. 2023.11.29.568895). bioRxiv. https://doi.org/10.1101/2023.11.29.568895

Ishihara, S. (1951). *Tests for colour-blindness*. Kanehara Tokyo.

Lüdecke, D., Ben-Shachar, M. S., Patil, I., Waggoner, P., & Makowski, D. (2021). performance: An R Package for Assessment, Comparison and Testing of Statistical Models. *Journal of Open Source Software*, *6*(60), 3139. https://doi.org/10.21105/joss.03139

Millard, S. P. (2013). *EnvStats: An R Package for Environmental Statistics*. Springer. https://www.springer.com

Stoet, G. (2010). PsyToolkit: A software package for programming psychological experiments using Linux. *Behavior Research Methods*, *42*, 1096–1104.

Stoet, G. (2017). PsyToolkit: A novel web-based method for running online questionnaires and reaction-time experiments. *Teaching of Psychology*, *44*(1), 24–31.
